# Supplementary material for: Total Synthesis and Anti-Inflammatory Bioactivity of (−)-Majusculoic Acid and Its Derivatives
Source: Mar Drugs. 2021 May 21;19(6):288. doi: 10.3390/md19060288 (PMC8223986; doi:10.3390/md19060288)

Supplementary Materials

Total Synthesis and Anti-inflammatory Bioactivity of (–)-Majusculoic Acid and its Derivatives

Hong-Xiu Xiao [1,2], Qing-Xiang Yan [2], Zhi-Hui He [2], Zheng-Biao Zou [2], Qing-Qing Le [2], Ting-Ting Chen [2], Bing Cai [2], Xian-Wen Yang [2,]* and Su-Lan Luo [1,]*

[1] Key Laboratory of Tropical Biological Resources of Ministry of Education, Key Laboratory for Marine Drugs of Haikou, School of Life and Pharmaceutical Sciences, Hainan University, Haikou 570228, China; xiaohongxiu97@hainanu.edu.cn

[2] Key Laboratory of Marine Biogenetic Resources, Third Institute of Oceanography, Ministry of Natural Resources,184 Daxue Road, Xiamen 361005, R. P. China; youngqx@126.com (Q.-X.Y.); hezhihui@tio.org.cn (Z.-H.H.); zhengbiaozou@njust.edu.cn (Z.-B.Z.); leqingqing@tio.org.cn (Q.-Q.L.); chentingting@tio.org.cn (T.-T.C.); caibing@tio.org.cn (B.C.)

***** Correspondence: e-mail: yangxianwen@tio.org.cn (X.-W.Y.); luosulan2003@163.com (S.-L.L.);

Table of Contents

1. Experimental Data S2

1.1. Total Synthesis of Majusculoic Acid S2

1.2. Synthesis of Majusculoic Acid Analogues..S4

2. NMR Spectra of Compounds S4

1. Experimental Data

1.1. Total Synthesis of Majusculoic Acid

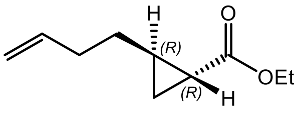

19

$[\alpha]_D^{25}$ = –45.3 (*c* 1.0, MeOH);

^{1}H NMR (400 MHz, $CDCl_3$) δ 5.78 (ddt, *J* = 17.0, 10.1, 6.7 Hz, 1H), 4.98 (dd, *J* = 17.1, 1.5 Hz, 1H), 4.93 (d, *J* = 10.2 Hz, 1H), 4.08 (q, *J* = 7.1 Hz, 2H), 2.20 – 2.04 (m, 2H), 1.44 – 1.28 (m, 4H), 1.22 (t, *J* = 7.1 Hz, 3H), 1.16 – 1.07 (m, 1H), 0.72 – 0.63 (m, 1H).

^{13}C NMR (100 MHz, $CDCl_3$) δ 174.3, 137.9, 114.8, 60.2, 33.2, 32.4, 22.3, 20.1, 15.3, 14.2;

IR (KBr, cm^{-1}) *v* 3445, 2980, 2922, 1728, 1514, 1203, 1178, 912;

HRMS (ESI, *m/z*) calcd for $C_{10}H_{16}O_2$ $[M+Na]^+$, 191.1048, found 191.1050.

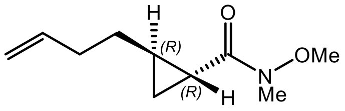

20

$[\alpha]_D^{25}$ = –25.2 (*c* 1.0, MeOH);

^{1}H NMR (400 MHz, $CDCl_3$) δ 5.56 (ddt, *J* = 16.9, 10.2, 6.7 Hz, 1H), 4.79 – 4.71 (m, 1H), 4.71 – 4.63 (m, 1H), 3.48 (s, 3H), 2.91 (s, 3H), 1.90 (dd, *J* = 14.6, 6.9 Hz, 2H), 1.70 – 1.60 (m, 1H), 1.17 (dd, *J* = 14.6, 7.1 Hz, 2H), 1.13 – 1.05 (m, 1H), 0.94 – 0.84 (m, 1H), 0.41 (ddd, *J* = 8.3, 6.0, 3.7 Hz, 1H).

^{13}C NMR (100 MHz, $CDCl_3$) δ 173.5, 137.3, 114.0, 77.2, 60.7, 32.7, 32.0, 21.2, 16.6, 14.3.

IR (KBr, cm^{-1}) *v* 2967, 1656, 1448, 1421, 1374, 1176, 1111, 998, 912;

HRMS (ESI, *m/z*) calcd for $C_{10}H_{17}NO_2$ $[M+Na]^+$, 206.1157, found 206.1170.

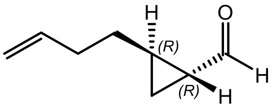

21

$[\alpha]_D^{25}$ = –10.6 (*c* 1.0, MeOH);

^{1}H NMR (400 MHz, $CDCl_3$) *δ* 8.94 (d, *J* = 5.5 Hz, 1H), 5.74 (ddt, *J* = 17.0, 10.2, 6.7 Hz, 1H), 4.96 (dd, *J* = 17.1, 1.6 Hz, 1H), 4.92 – 4.87 (m, 1H), 2.10 (q, *J* = 7.0 Hz, 2H), 1.62 – 1.52 (m, 1H), 1.48 – 1.33 (m, 3H), 1.29 – 1.16 (m, 1H), 0.92 – 0.83 (m, 1H).

^{13}C NMR (100 MHz, $CDCl_3$) *δ* 200.6, 137.5, 115.0, 33.1, 31.8, 30.2, 21.9, 14.6.

IR (KBr, cm^{-1}) *ν* 2961, 2925, 2854, 1688, 1261, 1082, 1019, 800;

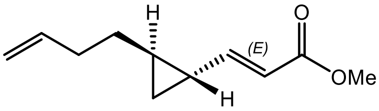

22

$[\alpha]_D^{25}$ = –59.0 (*c* 1.0, MeOH);

^{1}H NMR (400 MHz, $CDCl_3$) *δ* 6.46 (dd, *J* = 15.4, 10.1 Hz, 1H), 5.87 – 5.80 (d, 1H), 5.80 – 5.74 (ddt, 1H), 5.04 – 4.97 (m, 1H), 4.97 – 4.92 (m, 1H), 3.69 (s, 3H), 2.19 – 2.09 (m, 2H), 1.48 – 1.34 (m, 2H), 1.34 – 1.27 (m, 2H), 1.07 – 0.97 (m, 1H), 0.81 – 0.73 (m, 1H).

^{13}C NMR (100 MHz, $CDCl_3$) *δ* 167.2, 153.8, 138.1, 117.1, 114.8, 51.2, 33.4, 33.0, 22.8, 22.1, 16.0.

IR (KBr, cm^{-1}) *ν* 3000, 2921, 2852, 1721, 1647, 1434, 1266, 1148, 1053, 910, 843;

HRMS (ESI, *m/z*) calcd for $C_{11}H_{16}O_2$ $[M+Na]^+$, 203.1048, found 203.1050.

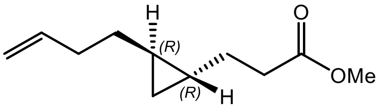

23

$[\alpha]_D^{25}$ = –6.7 (*c* 1.0, MeOH);

^{1}H NMR (400 MHz, $CDCl_3$) *δ* 5.87 – 5.72 (m, 1H), 5.02 – 4.94 (m, 1H), 4.91 (d, *J* = 10.2 Hz, 1H), 3.64 (s, 3H), 2.36 (t, *J* = 7.5 Hz, 2H), 2.09 (dd, *J* = 14.3, 7.3 Hz, 2H), 1.58 – 1.43 (m, 2H), 1.34 – 1.23 (m, 2H), 0.50 – 0.39 (m, 2H), 0.24 – 0.17 (m, 2H).

^{13}C NMR (100 MHz, $CDCl_3$) *δ* 174.1, 138.8, 114.3, 51.4, 34.2, 33.8, 33.5, 29.6, 18.4, 18.2, 11.8.

IR (KBr, cm^{-1}) *ν* 2921, 2852, 1747, 1680, 1196, 1141, 1079, 722;

HRMS (ESI, *m/z*) calcd for $C_{11}H_{18}O_2$ $[M+Na]^+$, 205.1204, found 205.1209.

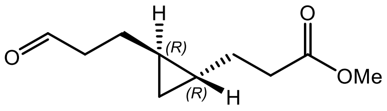

24

$[\alpha]_D^{25}$ = –8.9 (*c* 1.0, MeOH);

^{1}H NMR 1H NMR (400 MHz, $CDCl_3$) *δ* 9.73 (t, *J* = 1.8 Hz, 1H), 3.62 (s, 3H), 2.46 (td, *J* = 7.2, 1.7 Hz, 2H), 2.32 (t, *J* = 7.4 Hz, 2H), 1.53 – 1.43 (m, 4H), 0.50 – 0.40 (m, 2H), 0.22 (ddd, *J* = 8.8, 7.6, 3.9 Hz, 2H).

^{13}C NMR (100 MHz, $CDCl_3$) *δ* 202.5, 174.0, 51.4, 43.9, 34.1, 29.3, 26.7, 18.4, 18.1, 11.9.

IR (KBr, cm^{-1}) *v* 2992, 2926, 2857, 1738, 1713, 1440, 1195, 1179, 1027;

HRMS (ESI, *m/z*) calcd for $C_{10}H_{16}O_3$ $[M+Na]^+$, 207.0997, found 207.1003.

1.2. Synthesis of Majusculoic Acid Analogues

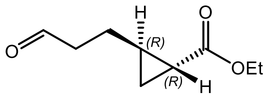

25

^{1}H NMR (400 MHz, $CDCl_3$) *δ* 9.70 (t, *J* = 1.3 Hz, 1H), 4.01 (q, *J* = 7.1 Hz, 2H), 2.49 (td, *J* = 7.2, 1.3 Hz, 2H), 1.64 – 1.42 (m, 2H), 1.36 – 1.23 (m, 2H), 1.15 (t, *J* = 7.1 Hz, 3H), 1.11 – 1.01 (m, 1H), 0.64 (ddd, *J* = 8.0, 6.5, 4.2 Hz, 1H).

^{13}C NMR (100 MHz, $CDCl_3$) *δ* 201.3, 173.7, 60.2, 43.1, 25.2, 21.6, 20.1, 15.1, 14.0.

IR (KBr, cm^{-1}) *v* 2983, 2936, 1724, 1649, 1450, 1412, 1269, 1179, 858;

HRMS (ESI, *m/z*) calcd for $C_9H_{14}O_3$ $[M+Na]^+$, 193.0841, found 193.0842.

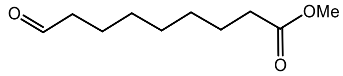

27

^{1}H NMR (400 MHz, $CDCl_3$) *δ* 9.74 (t, *J* = 1.6 Hz, 1H), 3.65 (s, 3H), 2.41 (td, *J* = 7.3, 1.5 Hz, 2H), 2.29 (t, *J* = 7.5 Hz, 2H), 1.60 (d, *J* = 4.9 Hz, 4H), 1.31 (s, 6H).

^{13}C NMR (100 MHz, $CDCl_3$) *δ* 202.8, 174.2, 51.5, 43.8, 34.0, 29.0, 28.9, 28.9, 24.8, 22.0.

2. NMR Spectra of Compounds

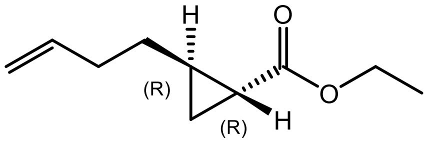

19

^{1}H NMR

(400 MHz, $CDCl_3$)

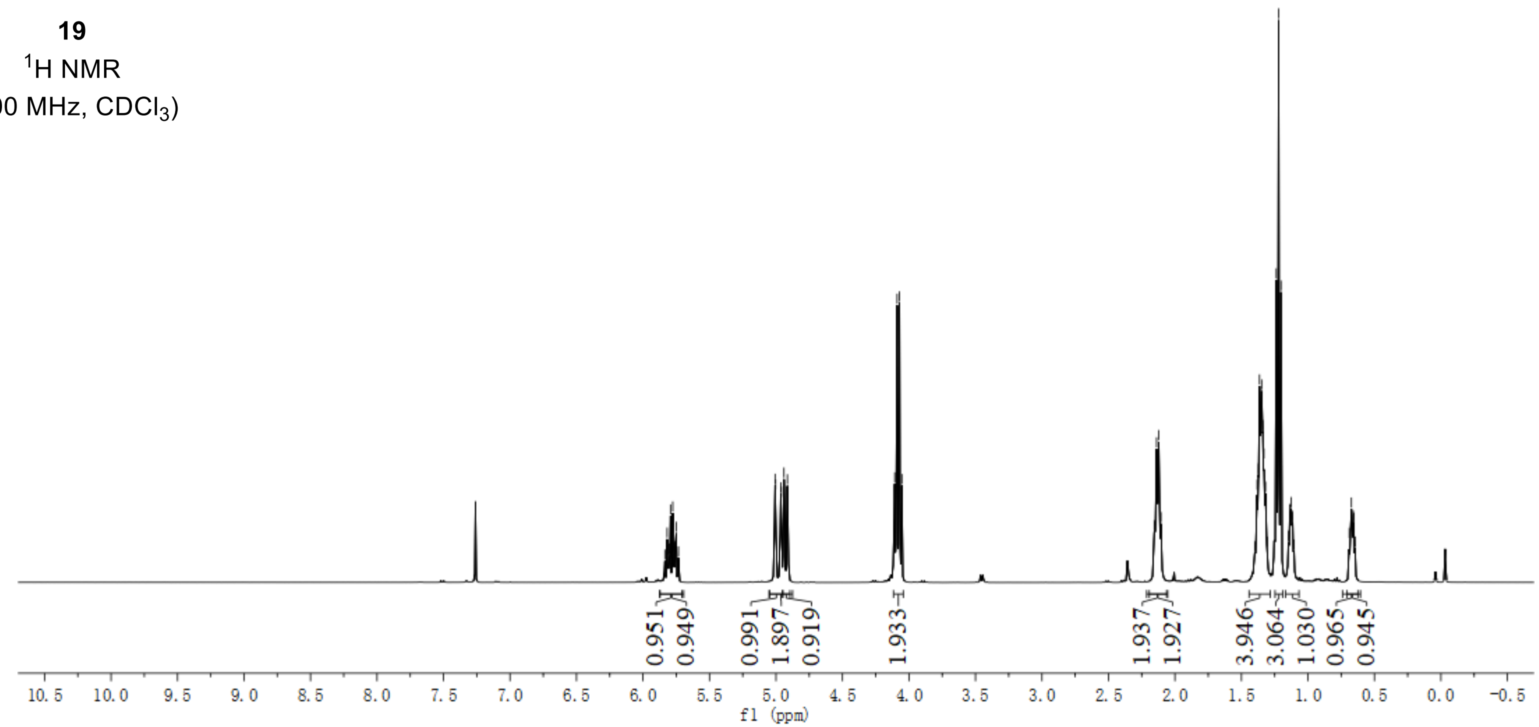

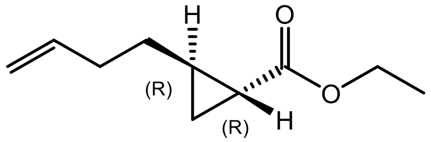

19

^{13}C NMR

(100 MHz, $CDCl_3$)

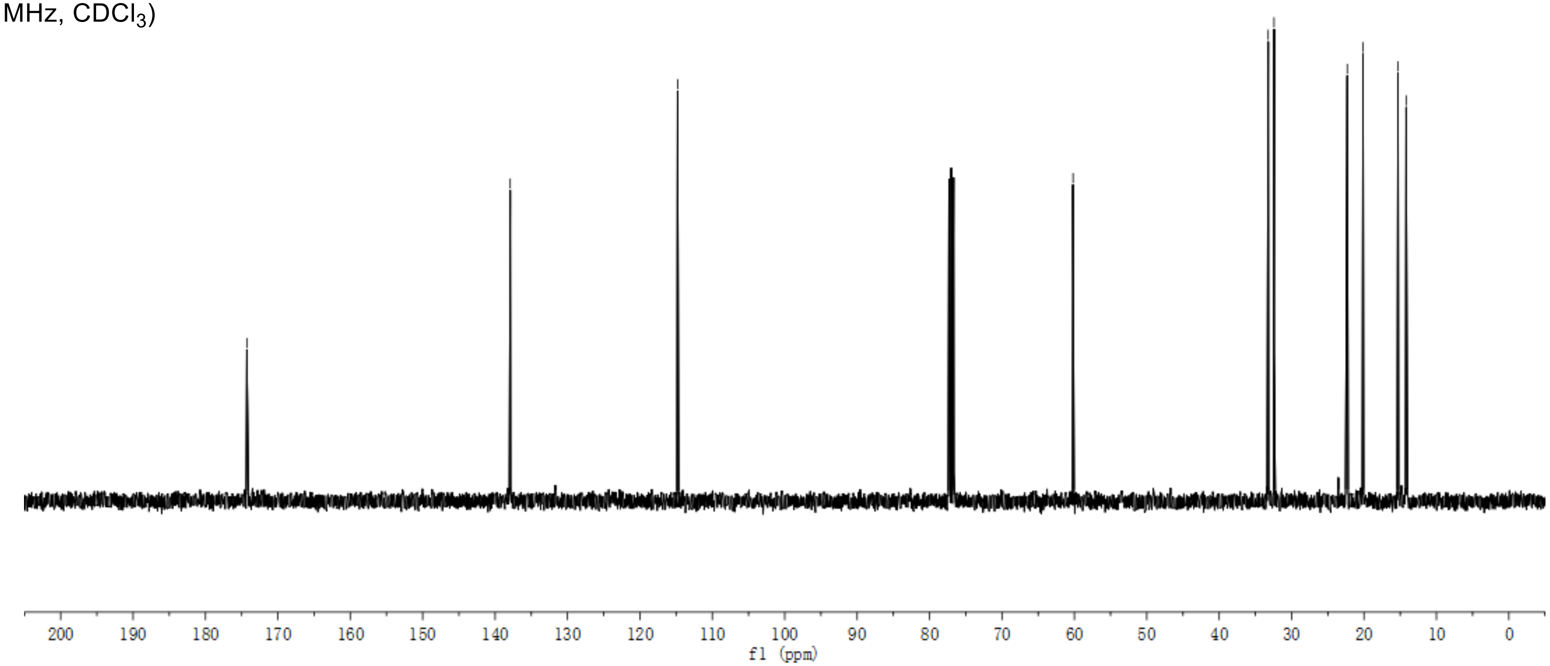

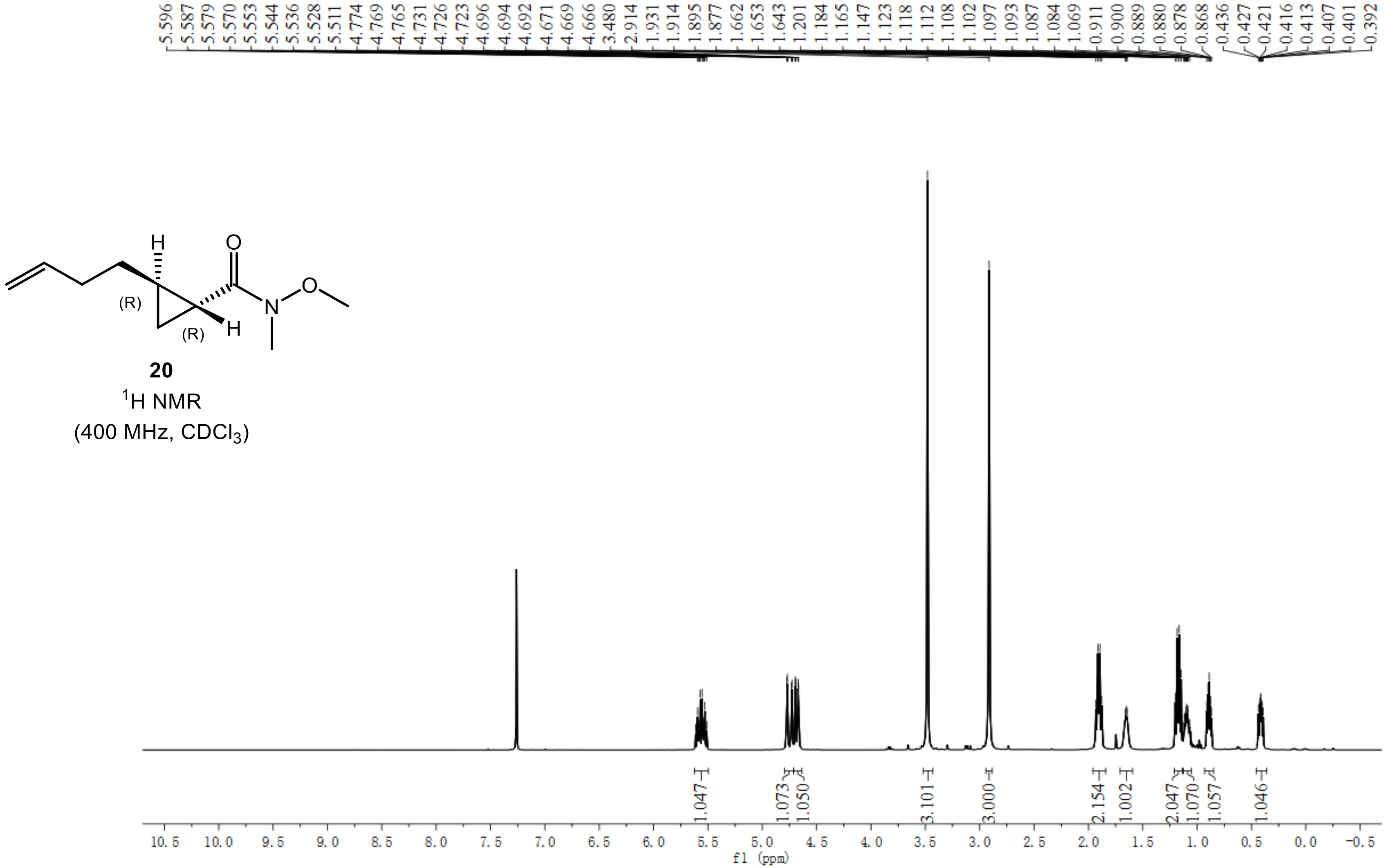

20

^{1}H NMR

(400 MHz, $CDCl_3$)

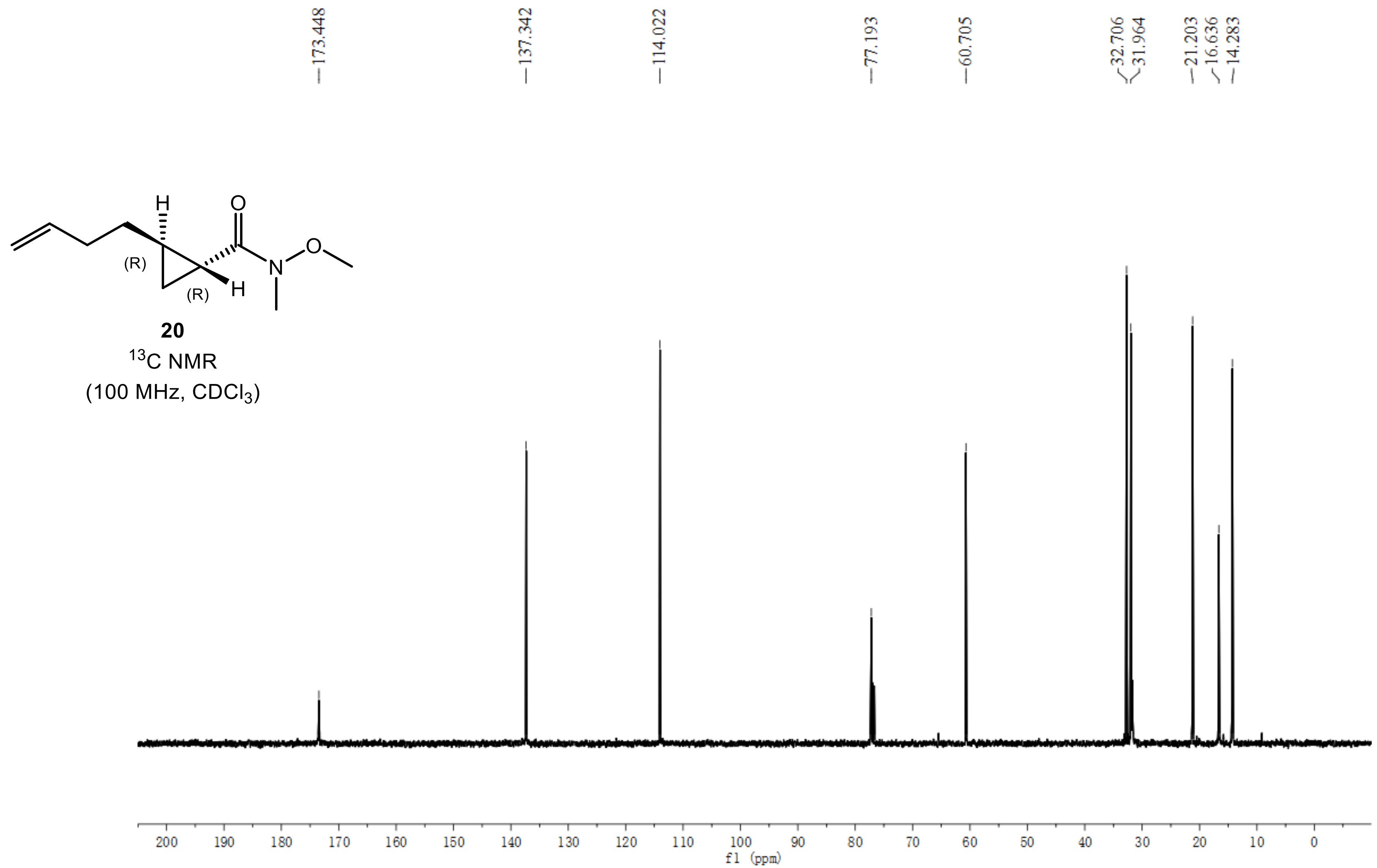

173.448
137.342
114.022
77.193
60.705
32.706
31.964
21.203
16.636
14.283
H
O
O
N
H
(R)
(R)
20
^{13}C NMR
(100 MHz, $CDCl_3$)
200
190
180
170
160
150
140
130
120
110
100
90
80
70
60
50
40
30
20
10
0
f1 (ppm)

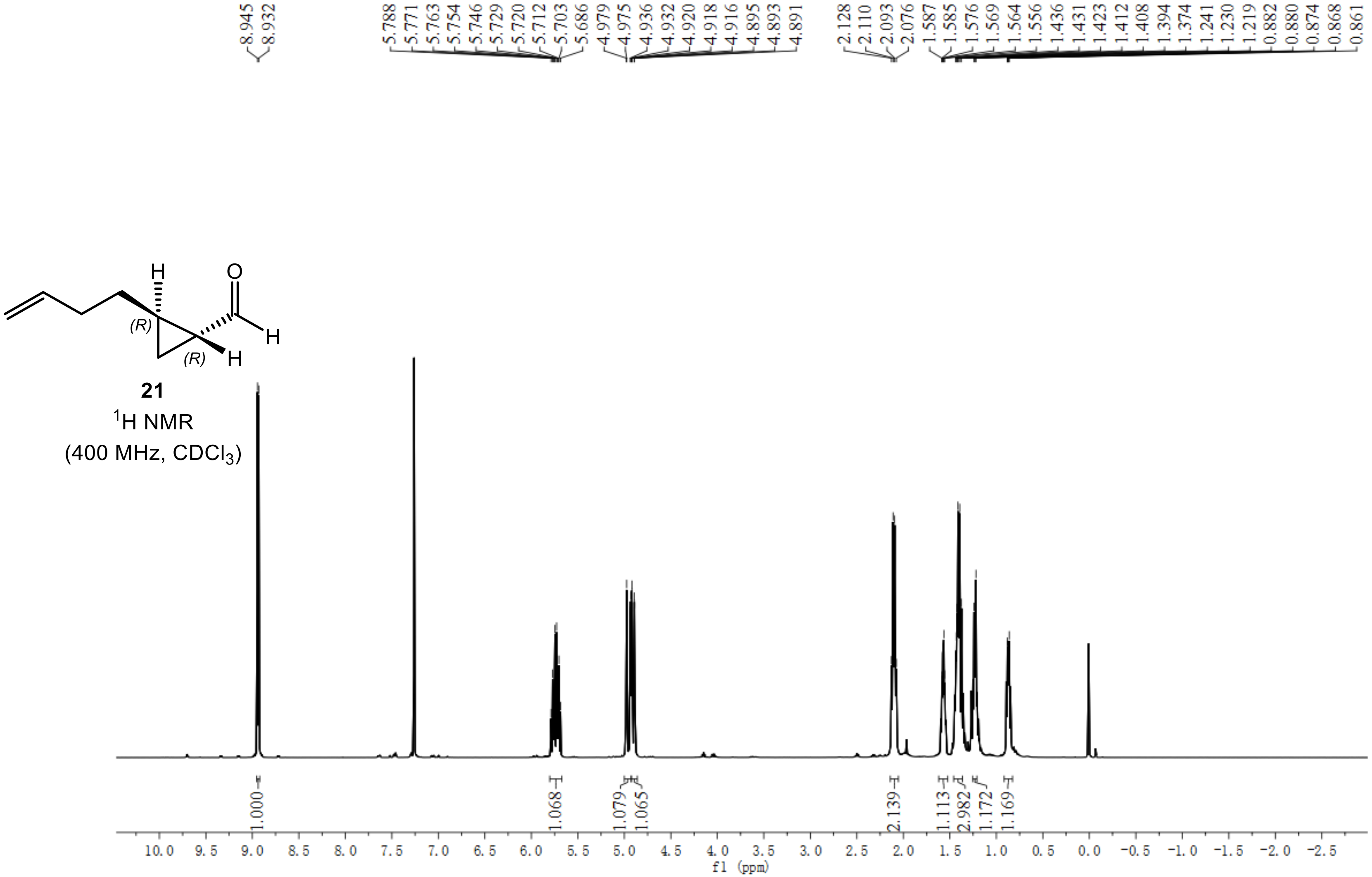

21

^1H NMR

(400 MHz, $CDCl_3$)

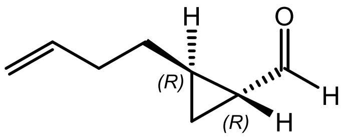

21

^{13}C NMR

(100 MHz, $CDCl_3$)

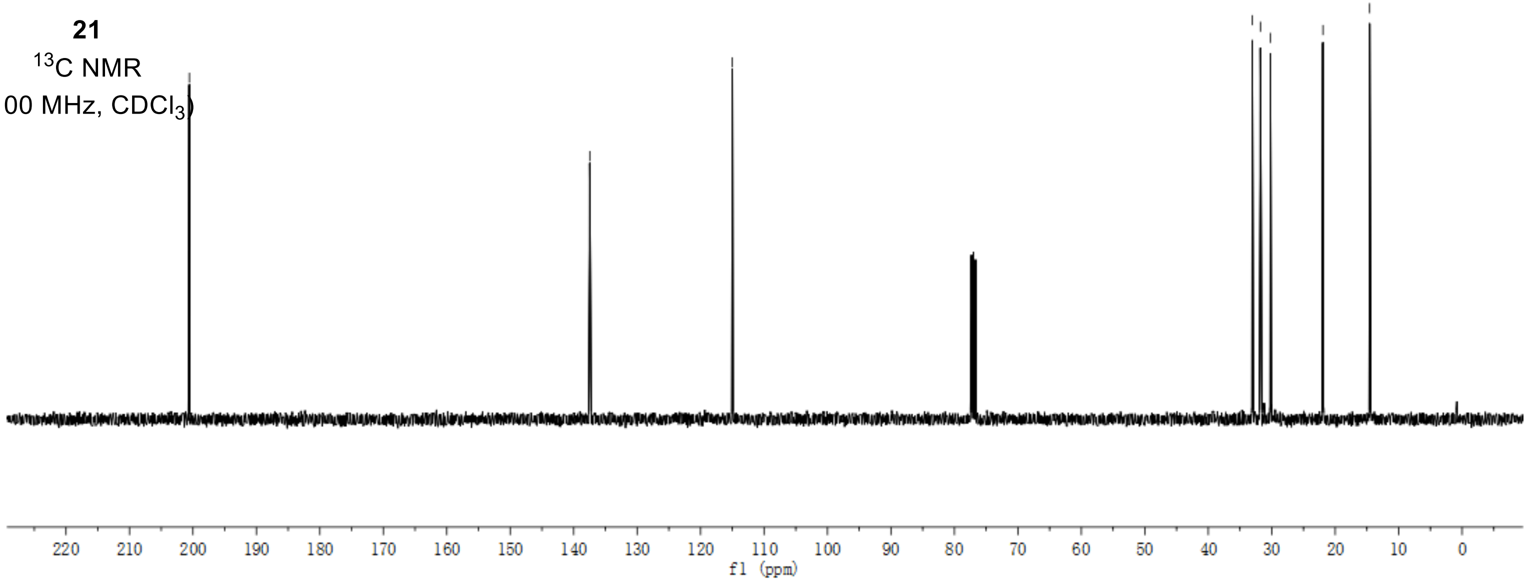

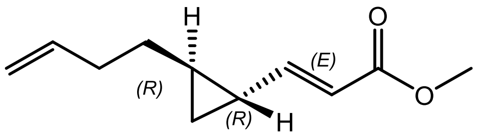

22

^{1}H NMR

(400 MHz, $CDCl_3$)

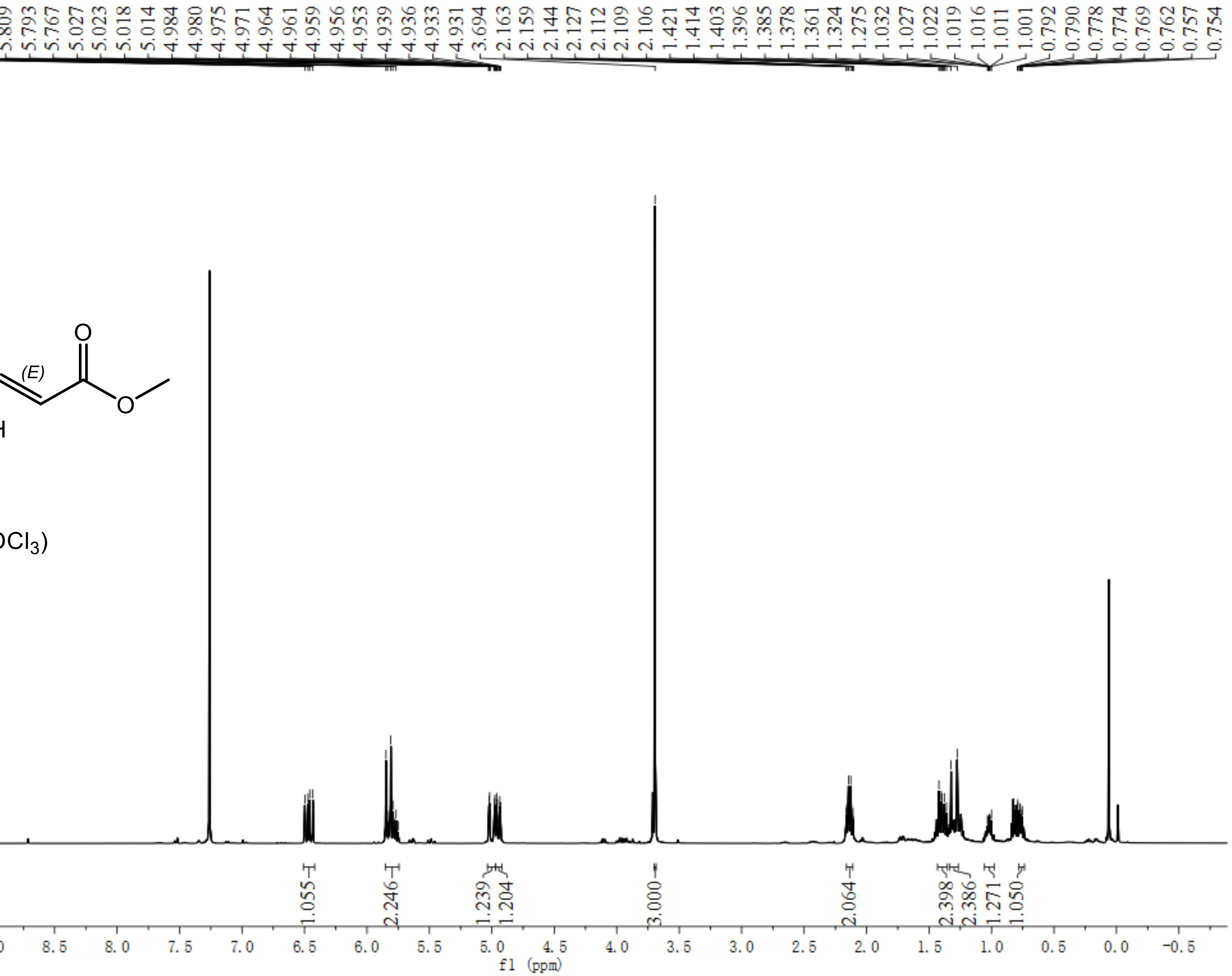

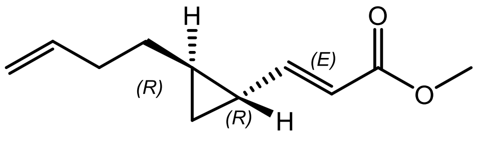

22
^{13}C NMR
(100 MHz, $CDCl_3$)

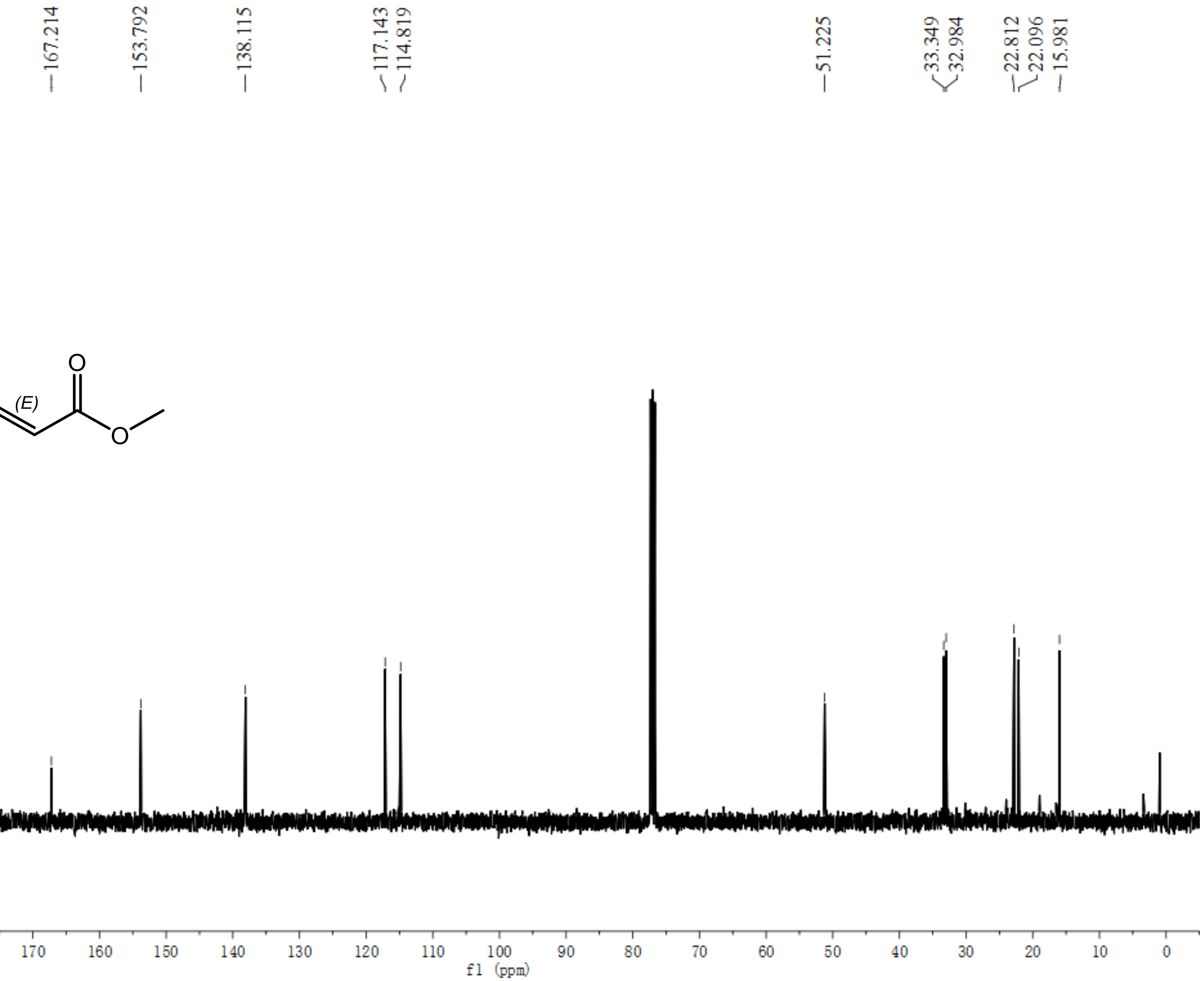

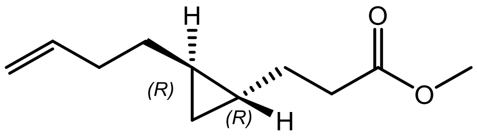

23

^{1}H NMR

(400 MHz, $CDCl_3$)

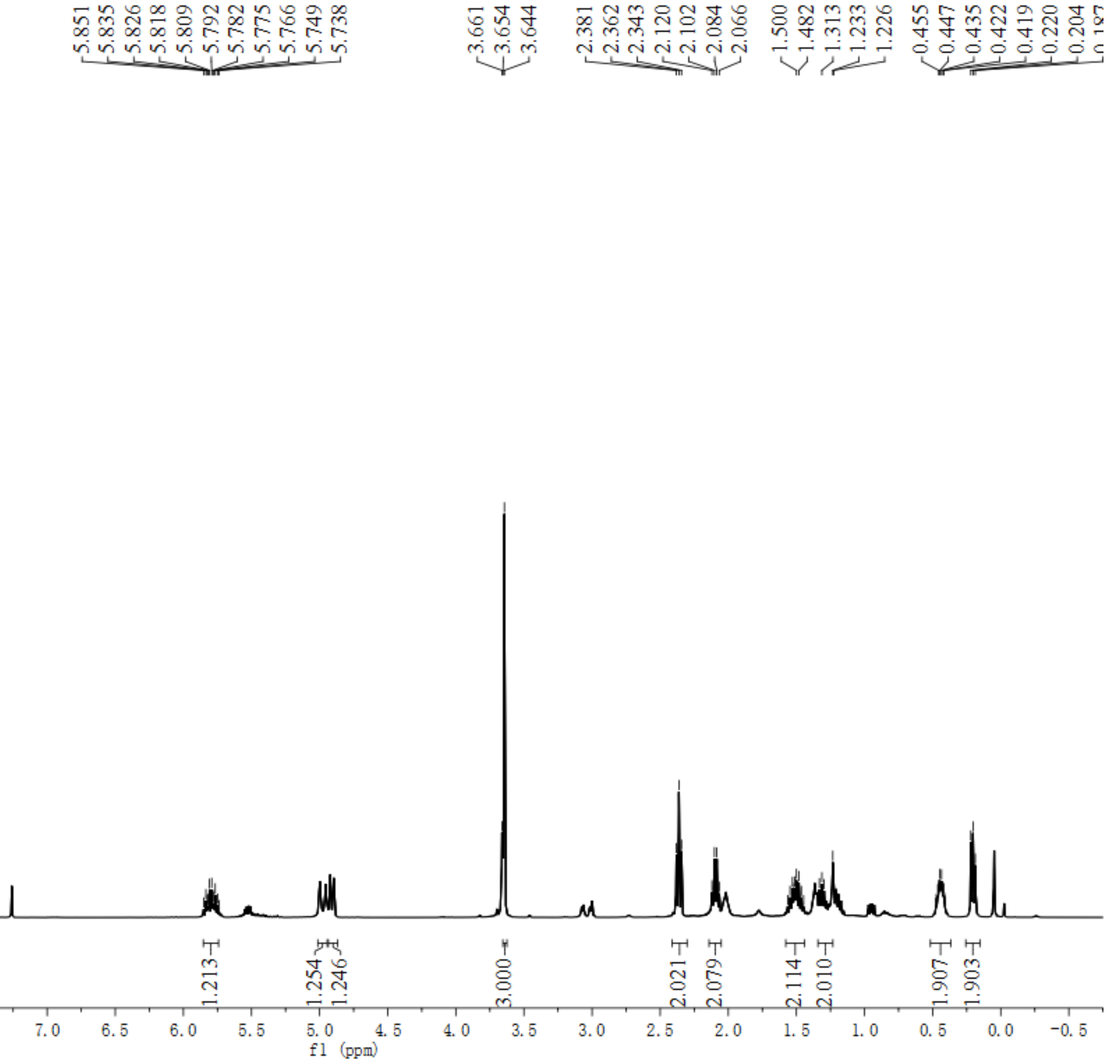

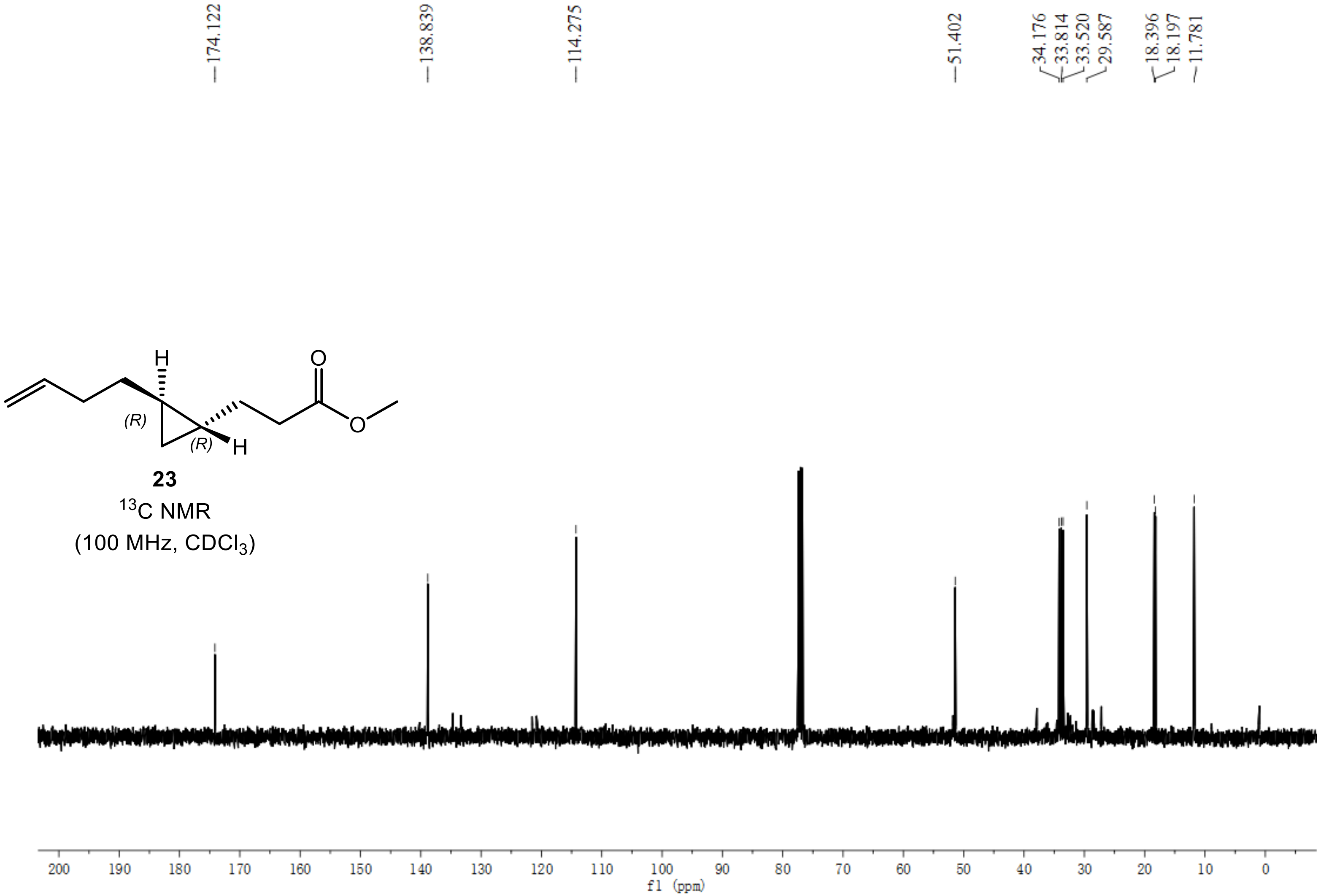

23

^{13}C NMR

(100 MHz, $CDCl_3$)

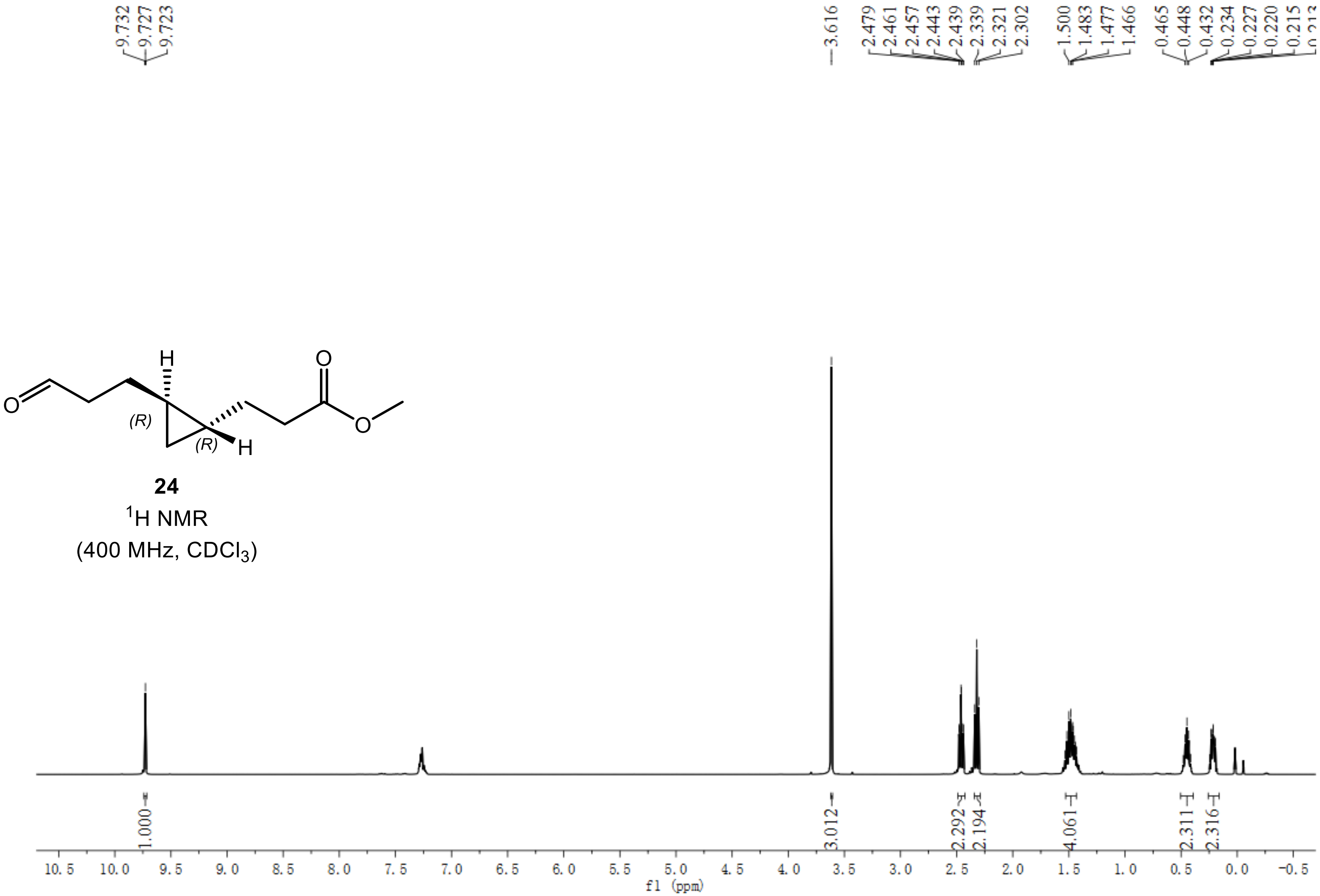

24

^{1}H NMR

(400 MHz, $CDCl_3$)

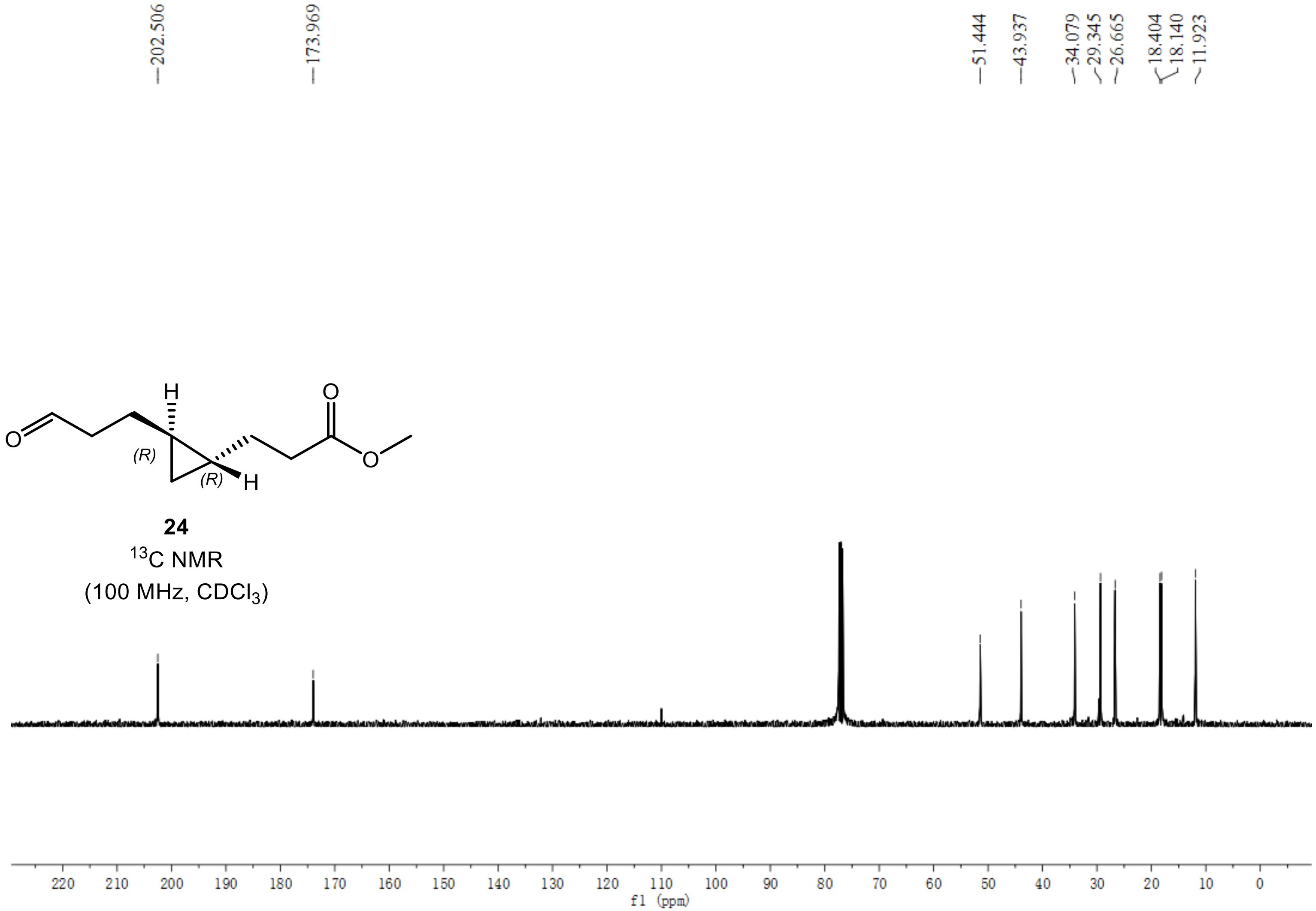

24

^{13}C NMR

(100 MHz, $CDCl_3$)

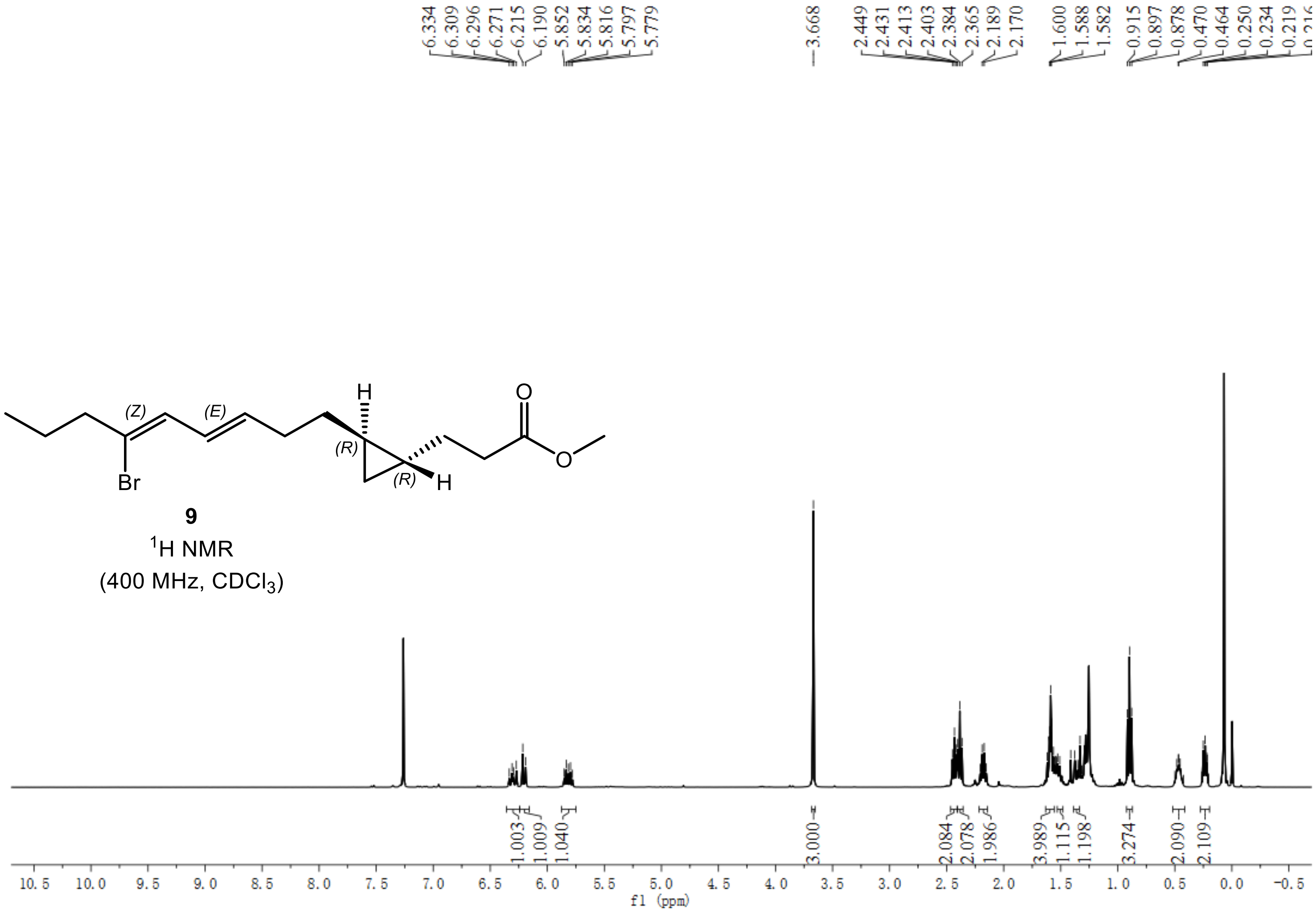

9

^{1}H NMR

(400 MHz, $CDCl_3$)

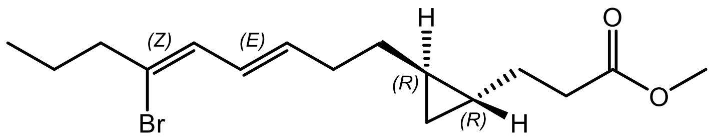

9

^{13}C NMR

(100 MHz, $CDCl_3$)

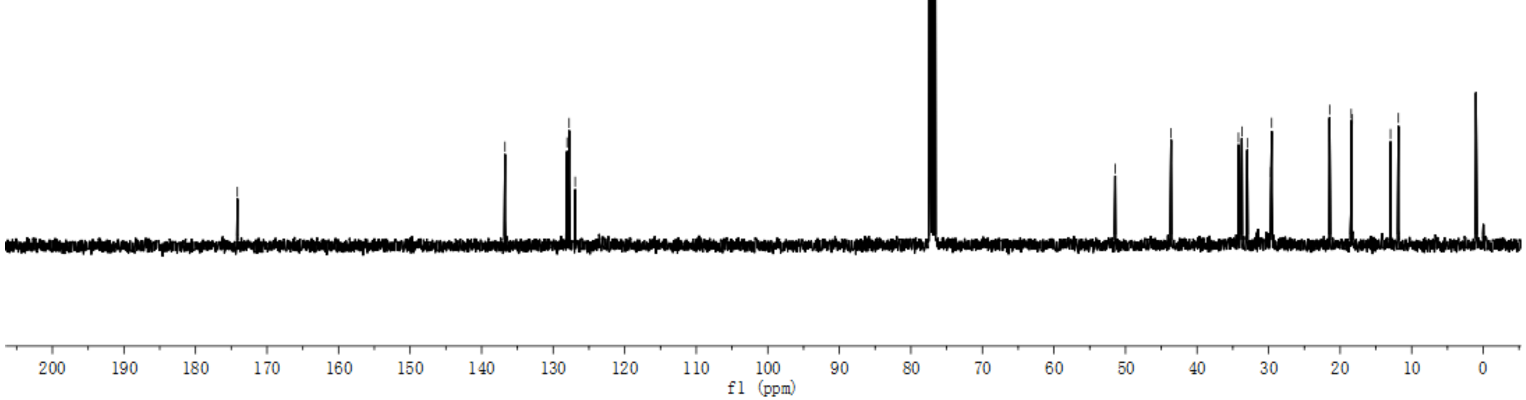

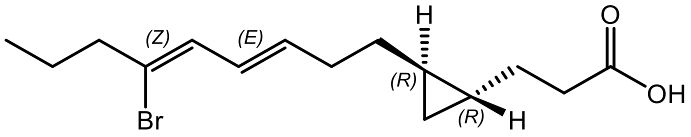

1

^{1}H NMR

(400 MHz, $CDCl_3$)

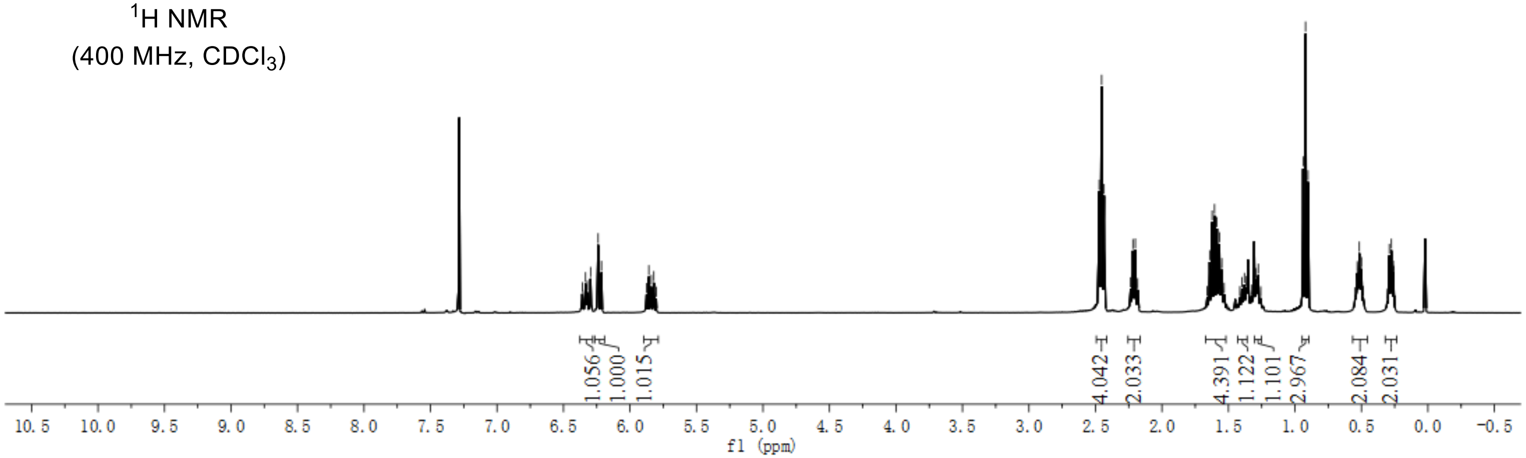

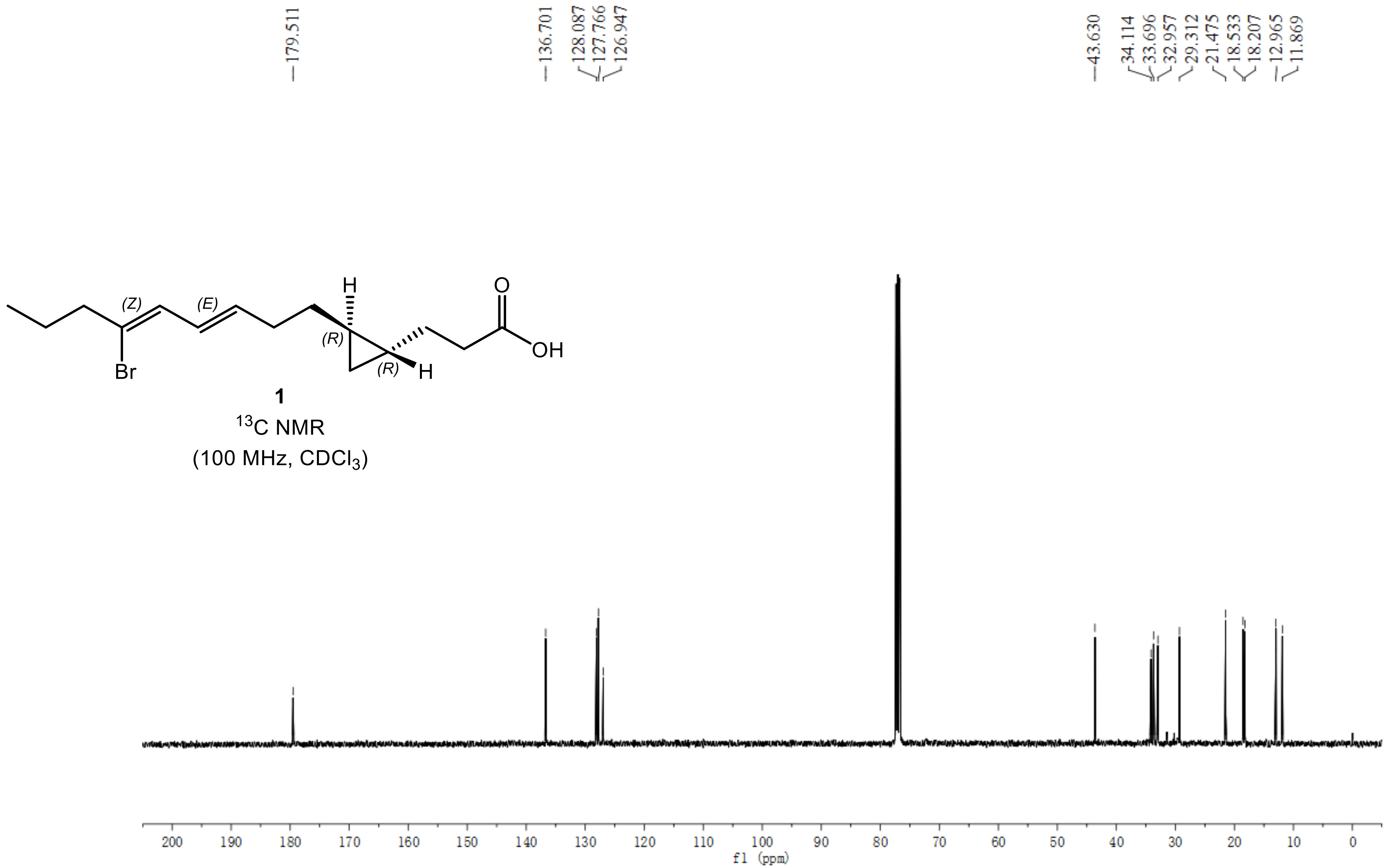

1

^{13}C NMR

(100 MHz, $CDCl_3$)

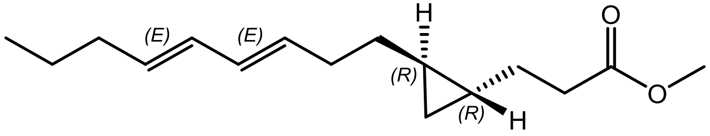

10

^{1}H NMR

(400 MHz, $CDCl_3$)

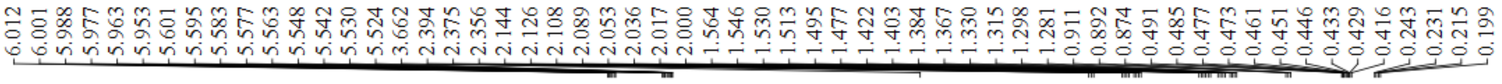
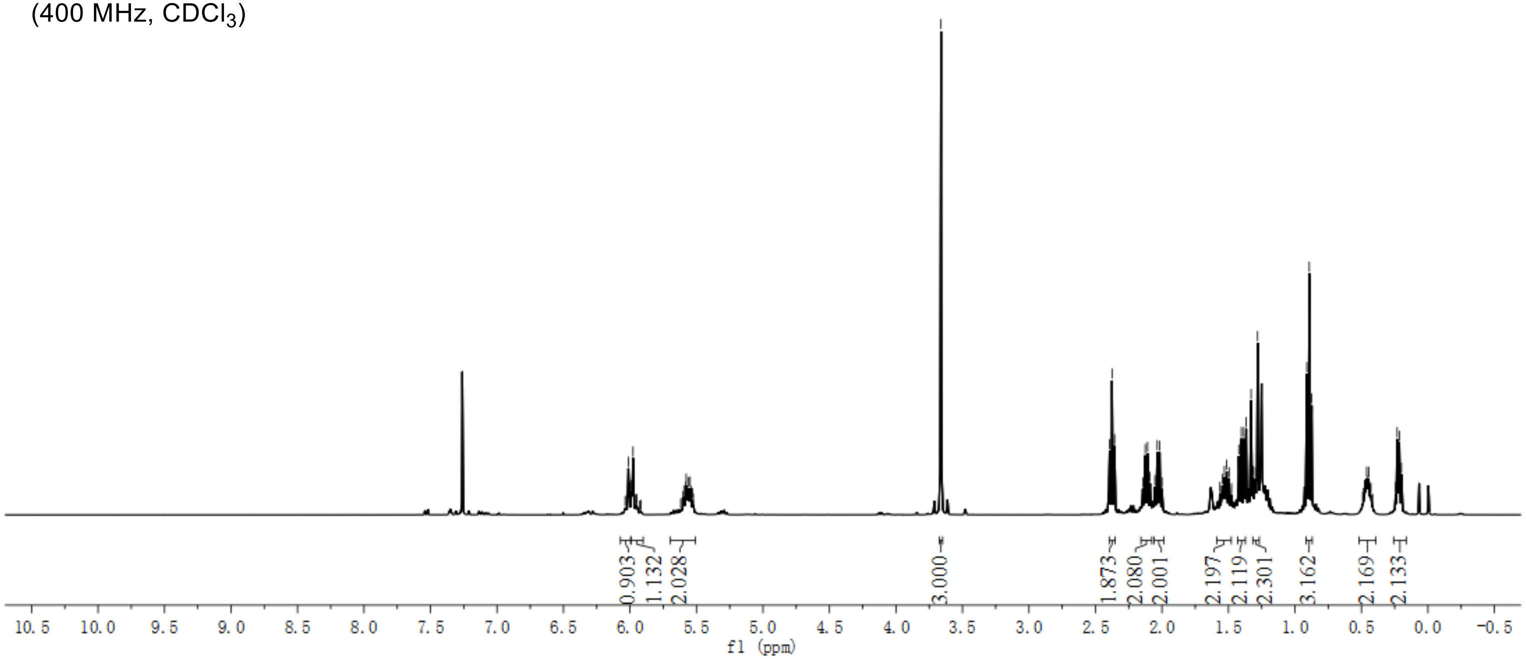

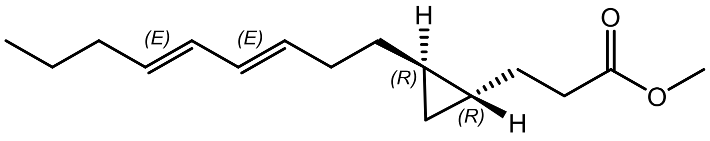

10

^{13}C NMR

(100 MHz, $CDCl_3$)

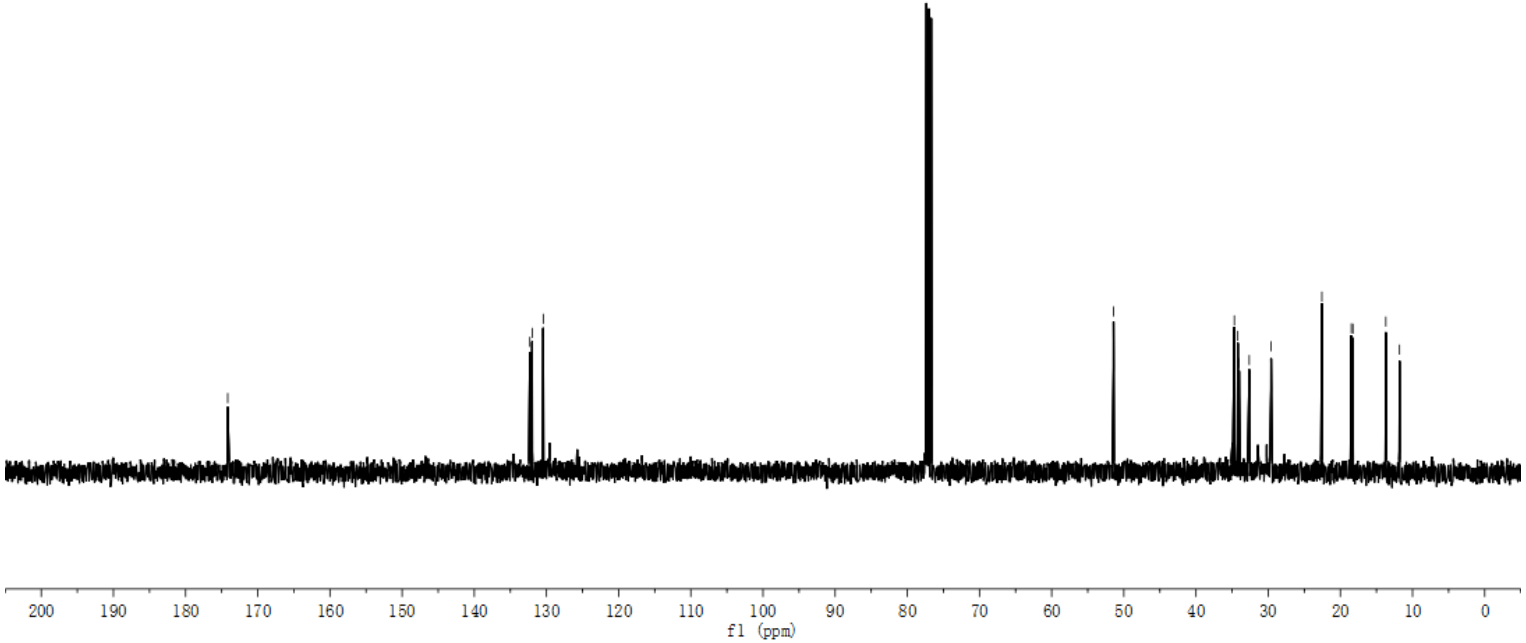

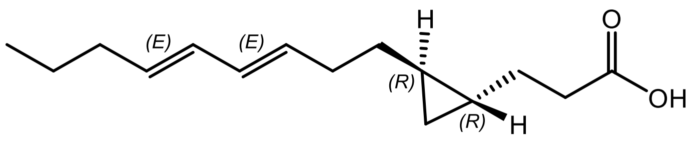

11

^{1}H NMR

(400 MHz, $CDCl_3$)

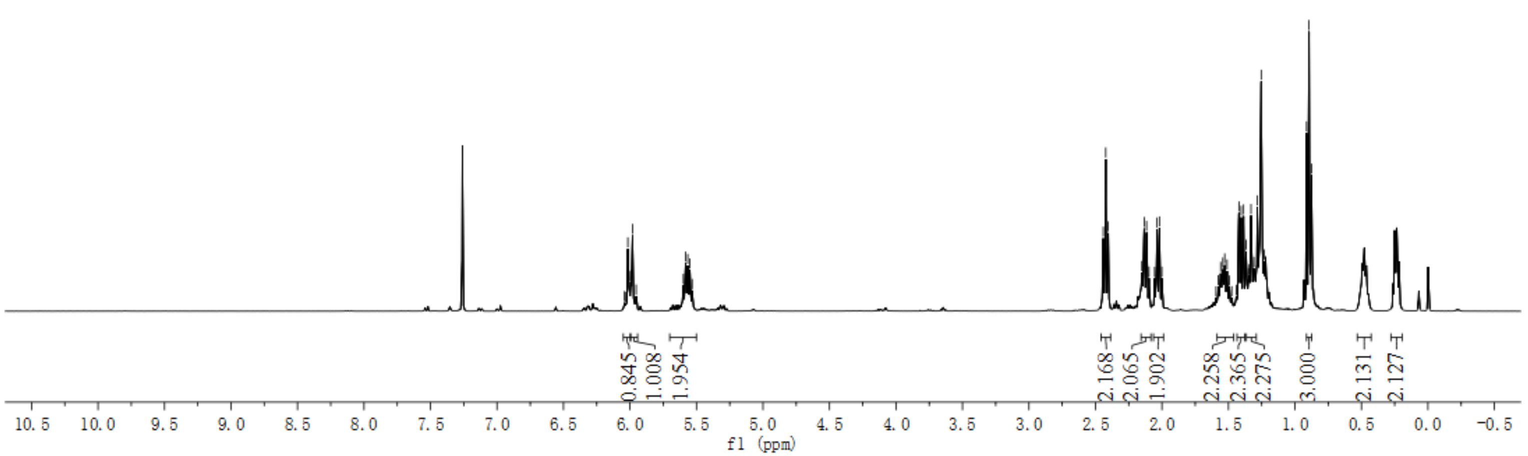

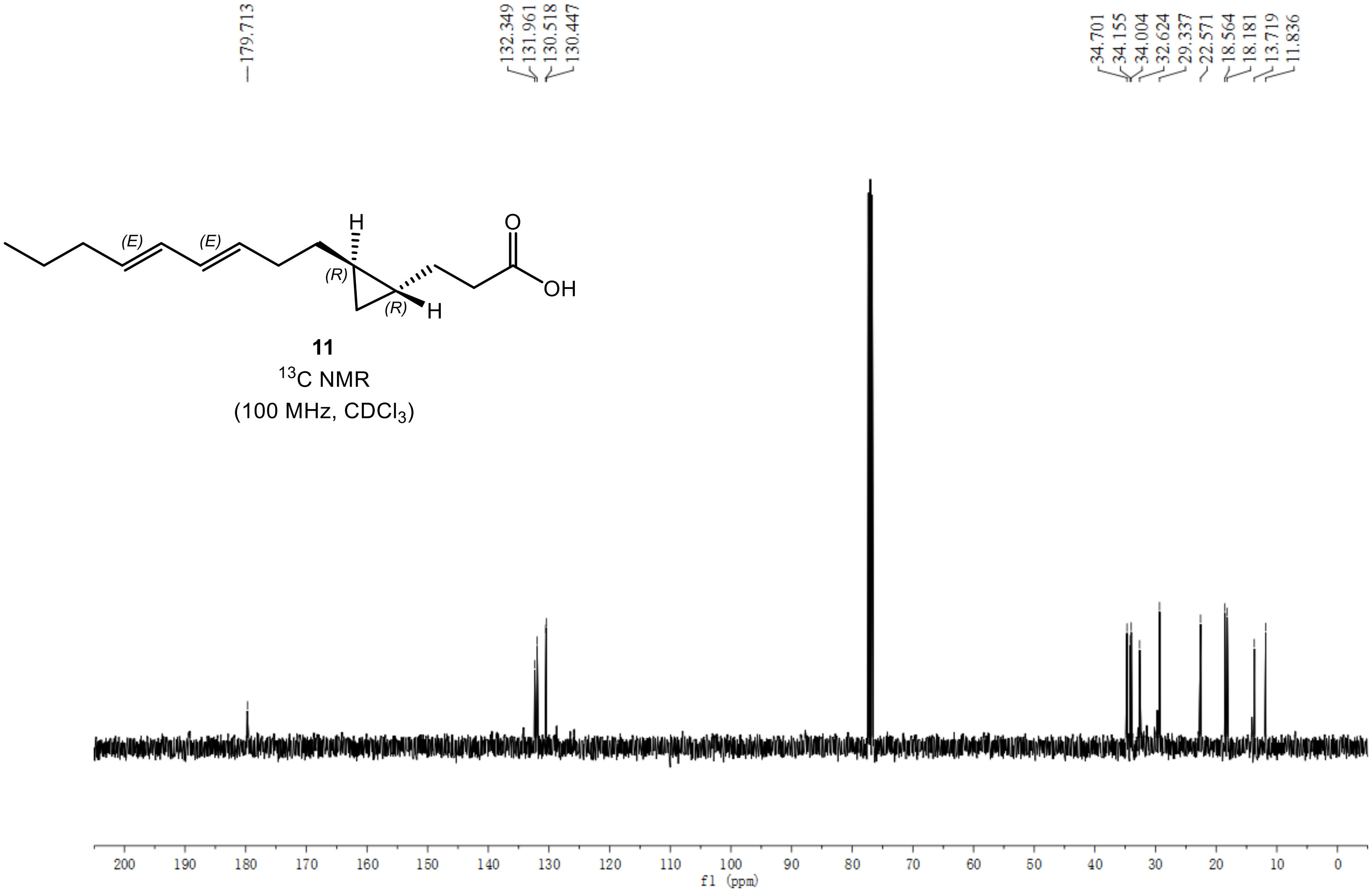

11

^{13}C NMR

(100 MHz, $CDCl_3$)

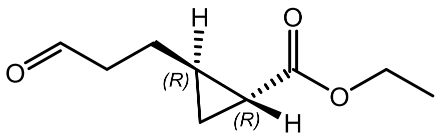

25
^{1}H NMR
(400 MHz, $CDCl_3$)

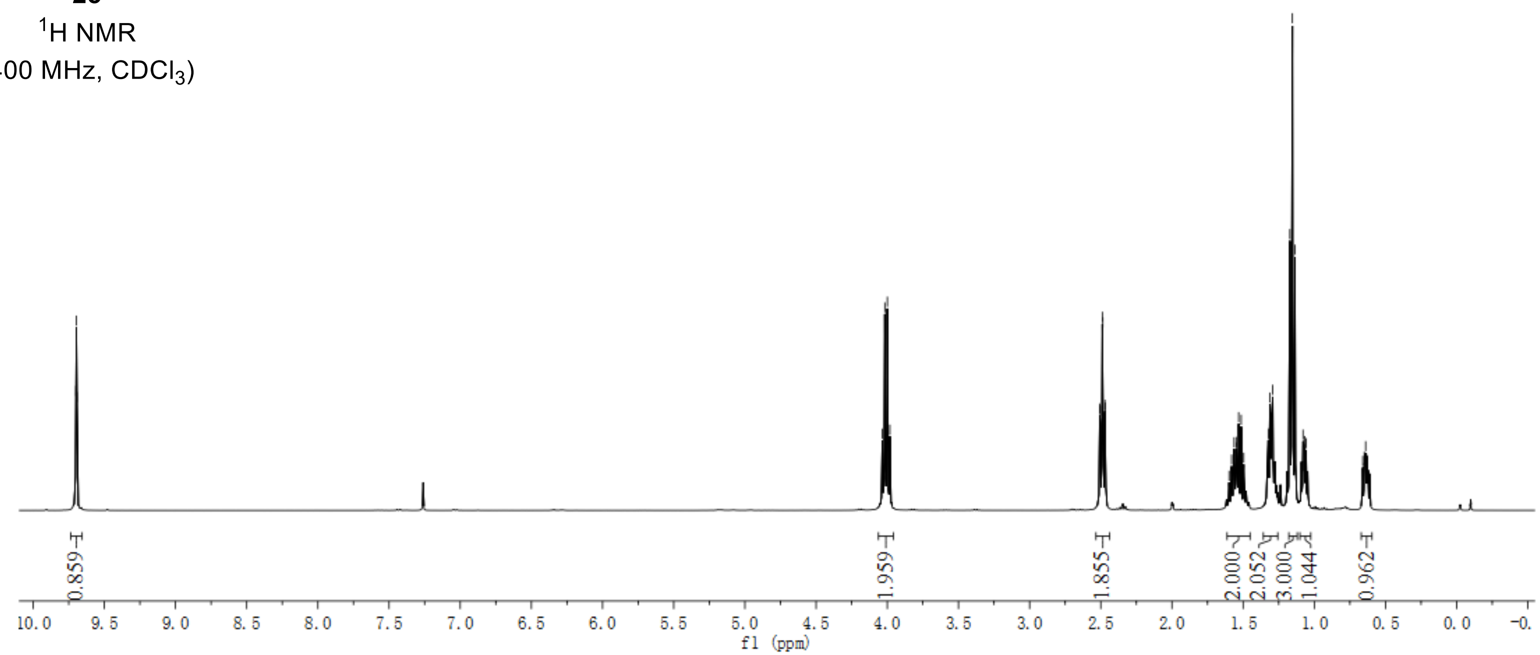

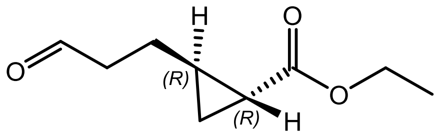

25

^{13}C NMR

(100 MHz, $CDCl_3$)

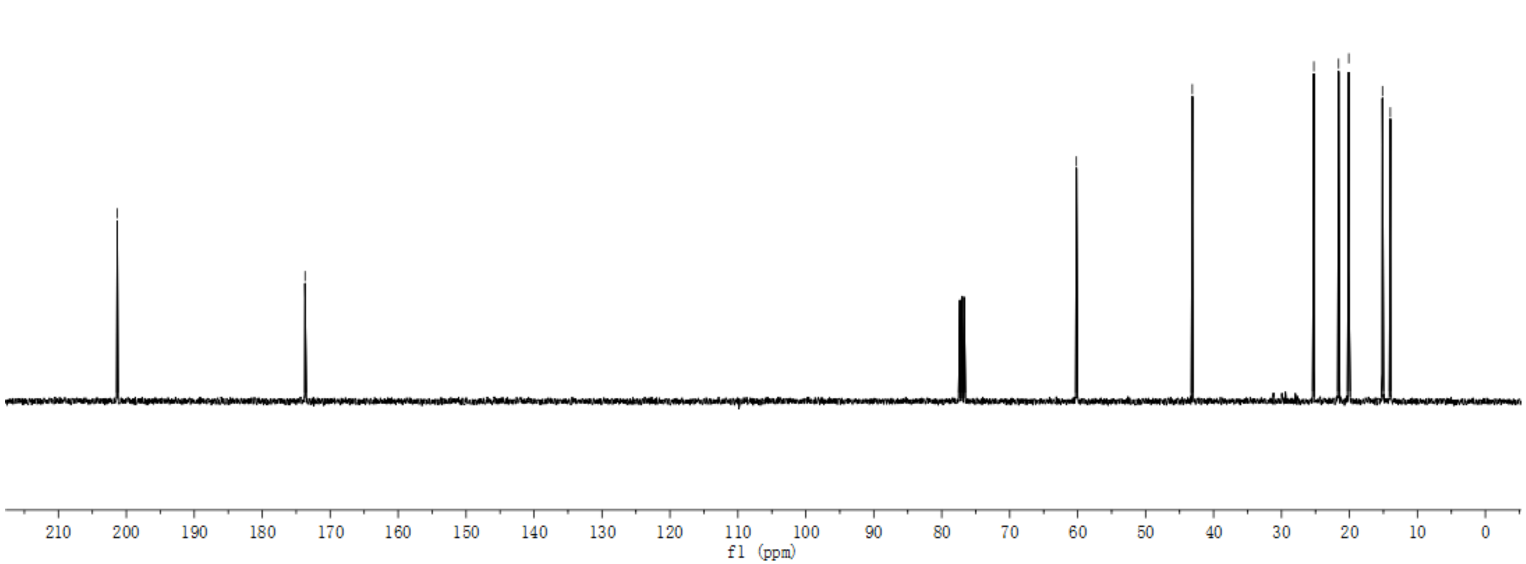

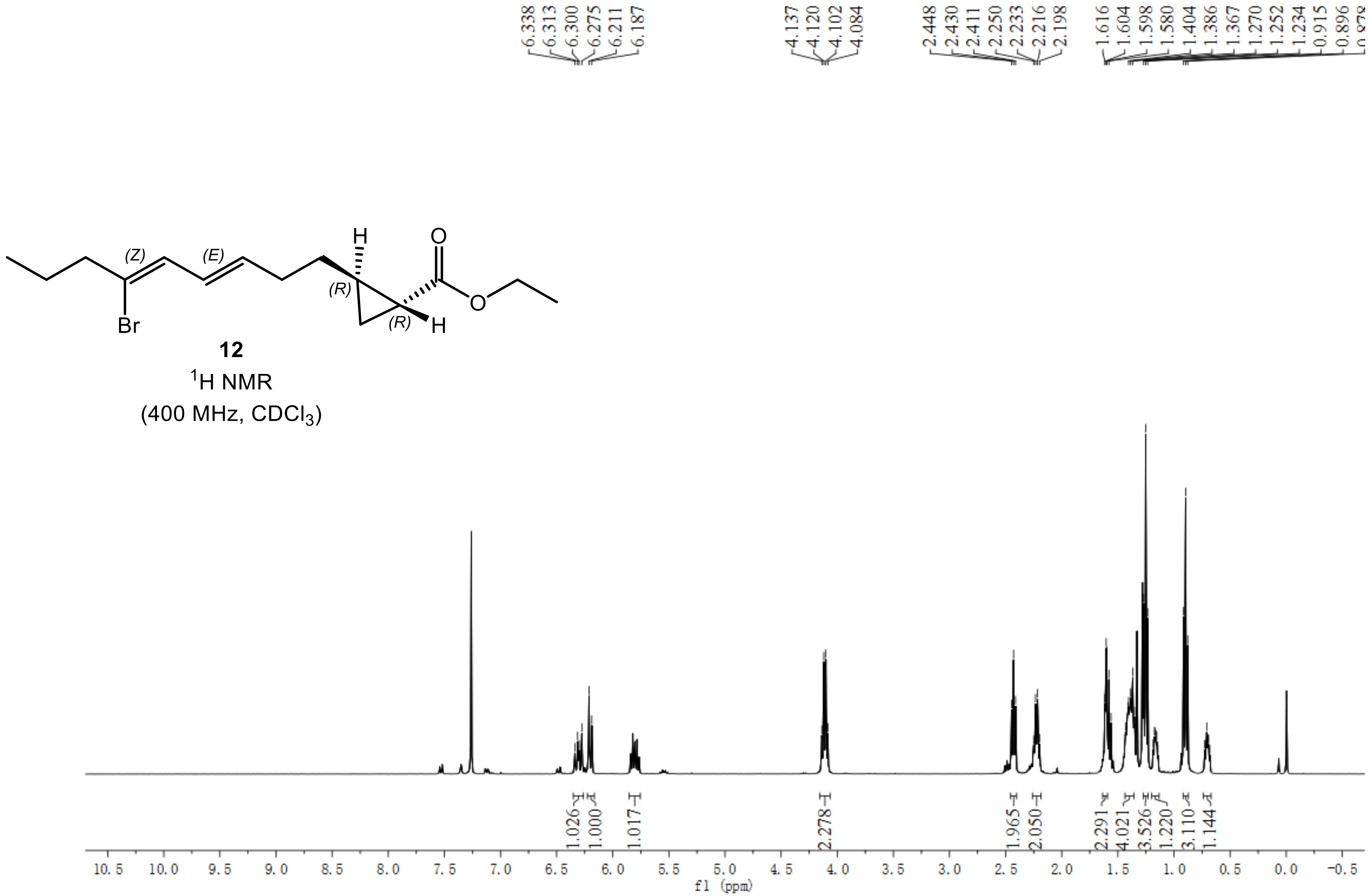

(Z)
(E)
H
O
(R)
O
(R)
H
Br
12
1H NMR
(400 MHz, CDCl3)
6.338
6.313
6.300
6.275
6.211
6.187
4.137
4.120
4.102
4.084
2.448
2.430
2.411
2.250
2.233
2.216
2.198
1.616
1.604
1.598
1.580
1.404
1.386
1.367
1.270
1.252
1.234
0.915
0.896
1.026
1.000
1.017
2.278
1.965
2.050
2.291
4.021
3.526
1.220
3.110
1.144
10.5
10.0
9.5
9.0
8.5
8.0
7.5
7.0
6.5
6.0
5.5
5.0
4.5
4.0
3.5
3.0
2.5
2.0
1.5
1.0
0.5
0.0
-0.5
f1 (ppm)

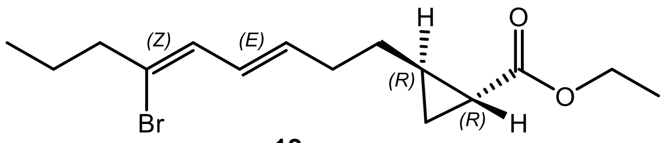

12

^{13}C NMR

(100 MHz, $CDCl_3$)

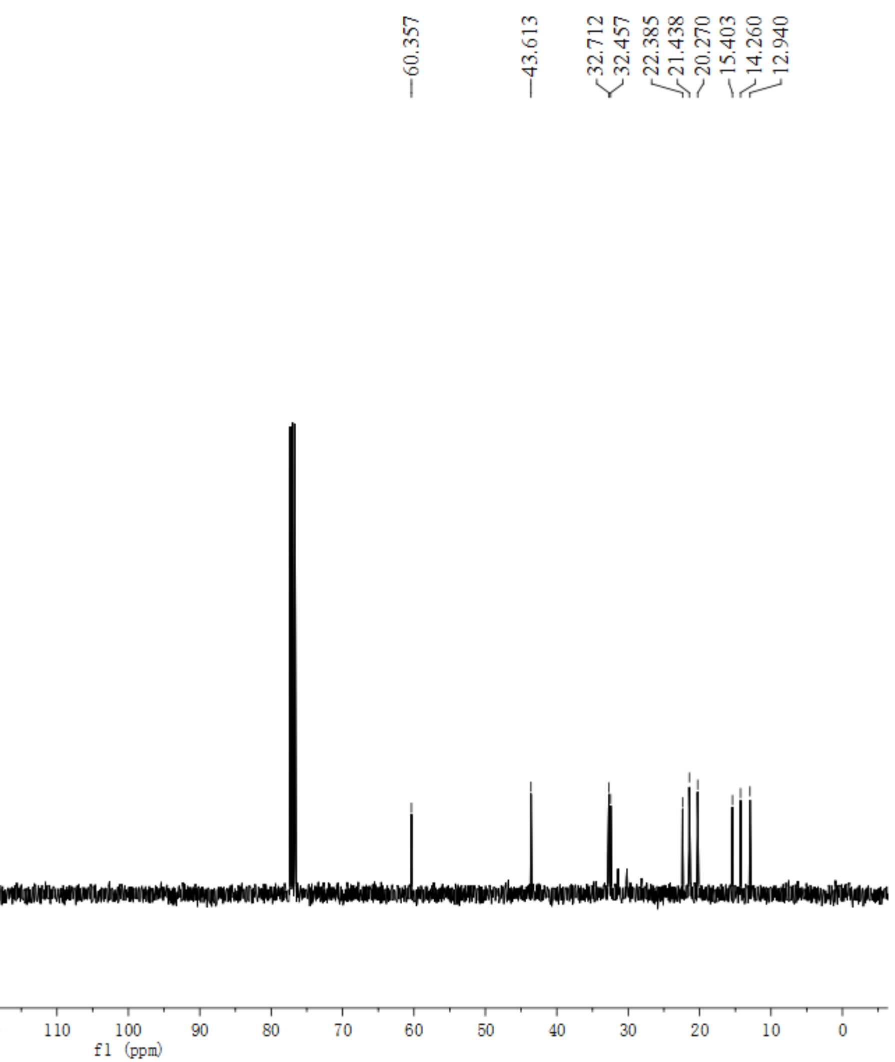

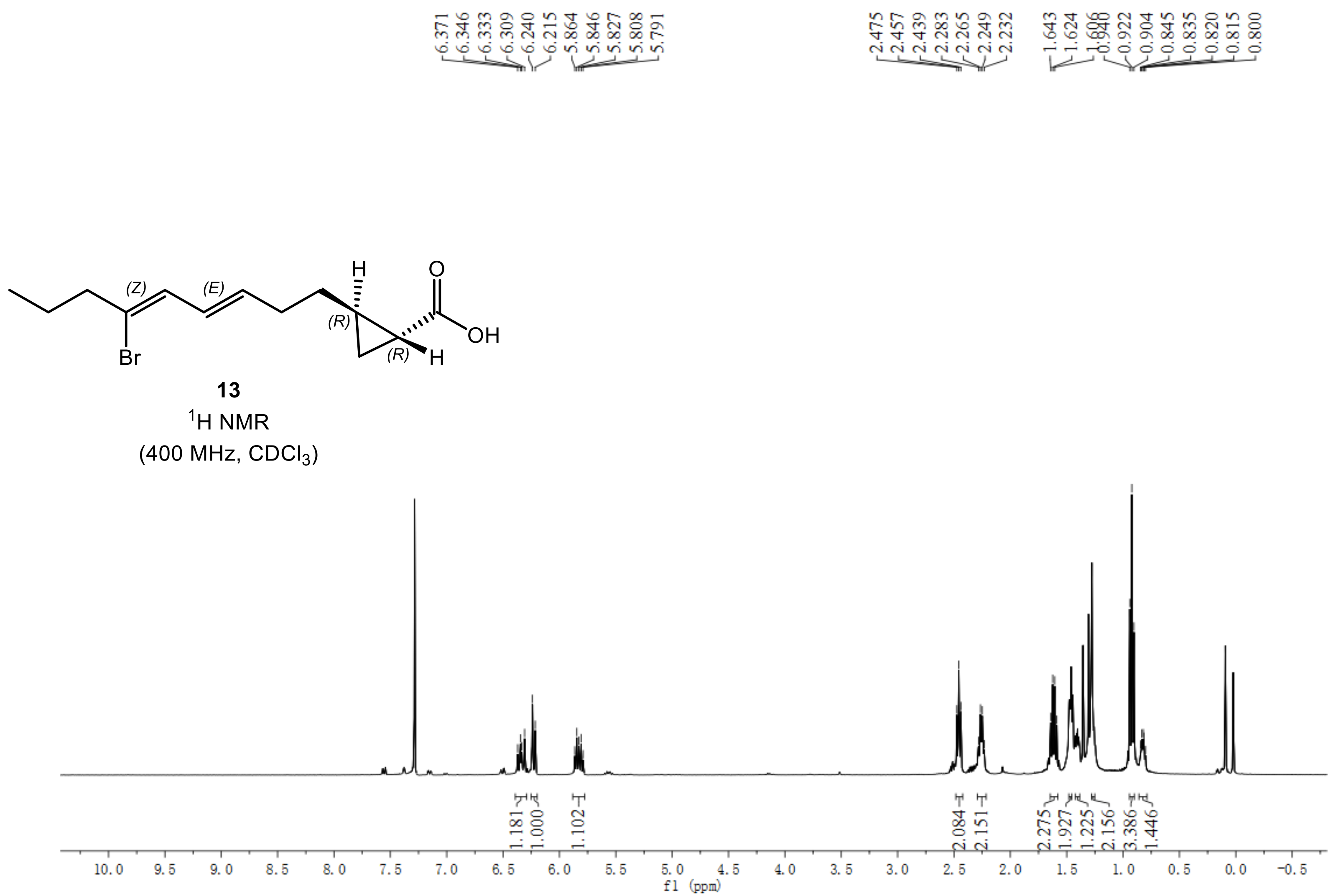

(Z)
(E)
(R)
(R)
H
O
OH
H
Br
13
1H NMR
(400 MHz, CDCl3)
6.371
6.346
6.333
6.309
6.240
6.215
5.864
5.846
5.827
5.808
5.791
2.475
2.457
2.439
2.283
2.265
2.249
2.232
1.643
1.624
1.606
0.940
0.922
0.904
0.845
0.835
0.820
0.815
0.800
1.181
1.000
1.102
2.084
2.151
2.275
1.927
1.225
2.156
3.386
1.446
f1 (ppm)

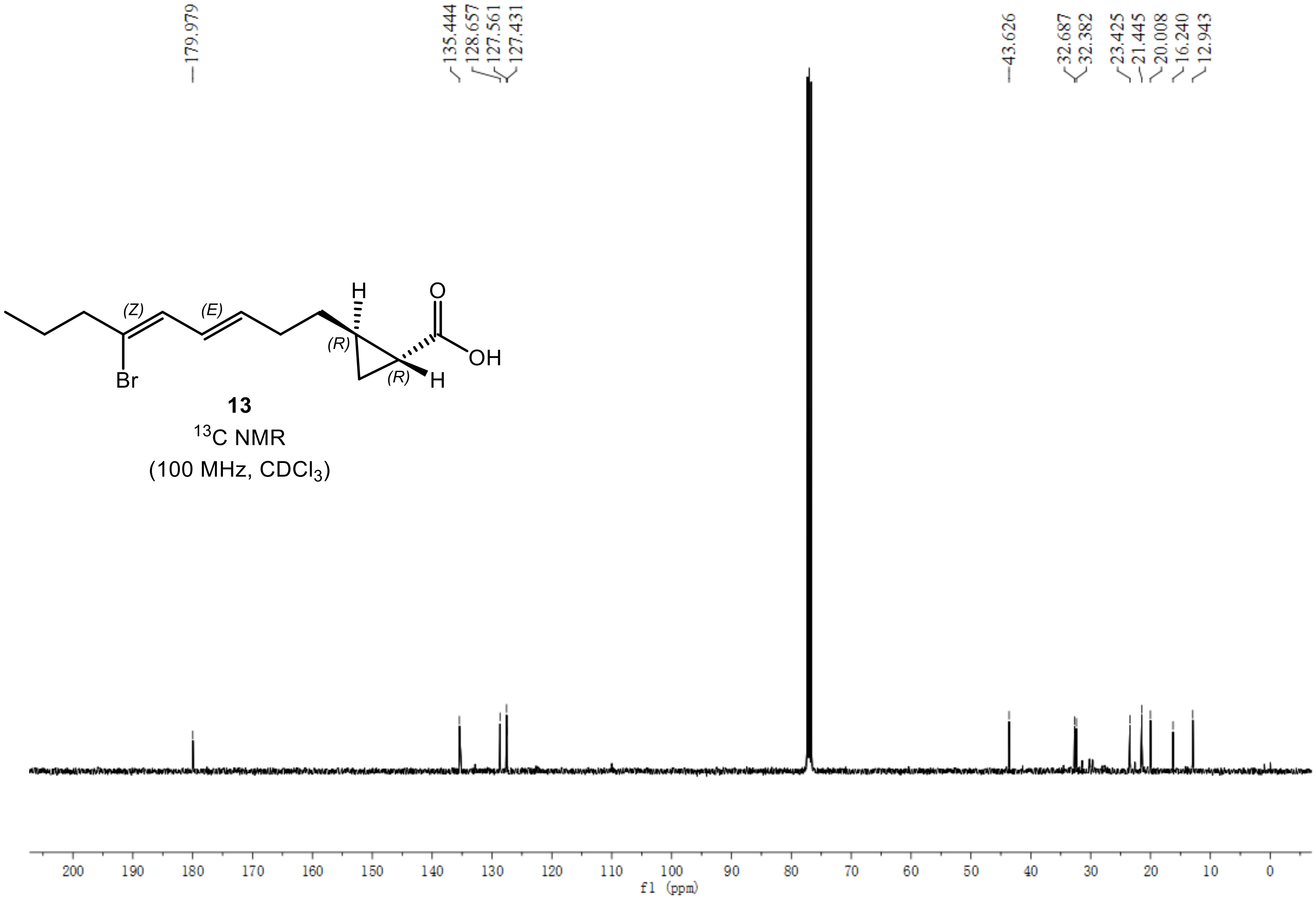

13

^{13}C NMR

(100 MHz, $CDCl_3$)

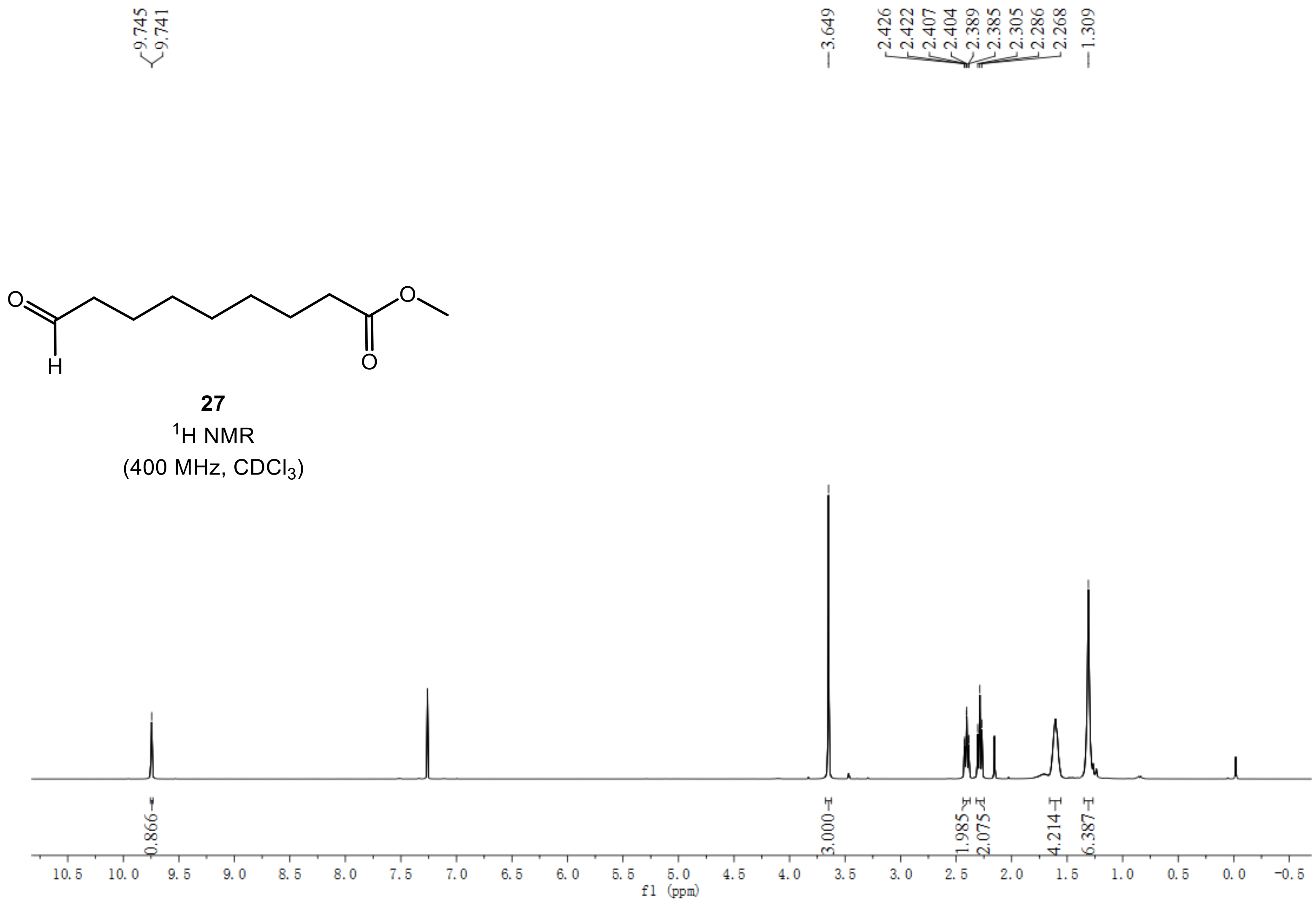
9.745
9.741
3.649
2.426
2.422
2.407
2.404
2.389
2.385
2.305
2.286
2.268
1.309
27
^{1}H NMR
(400 MHz, $CDCl_3$)
0.866
3.000
1.985
2.075
4.214
6.387
10.5 10.0 9.5 9.0 8.5 8.0 7.5 7.0 6.5 6.0 5.5 5.0 4.5 4.0 3.5 3.0 2.5 2.0 1.5 1.0 0.5 0.0 -0.5
f1 (ppm)

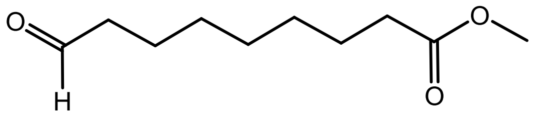

27

^{13}C NMR

(100 MHz, $CDCl_3$)

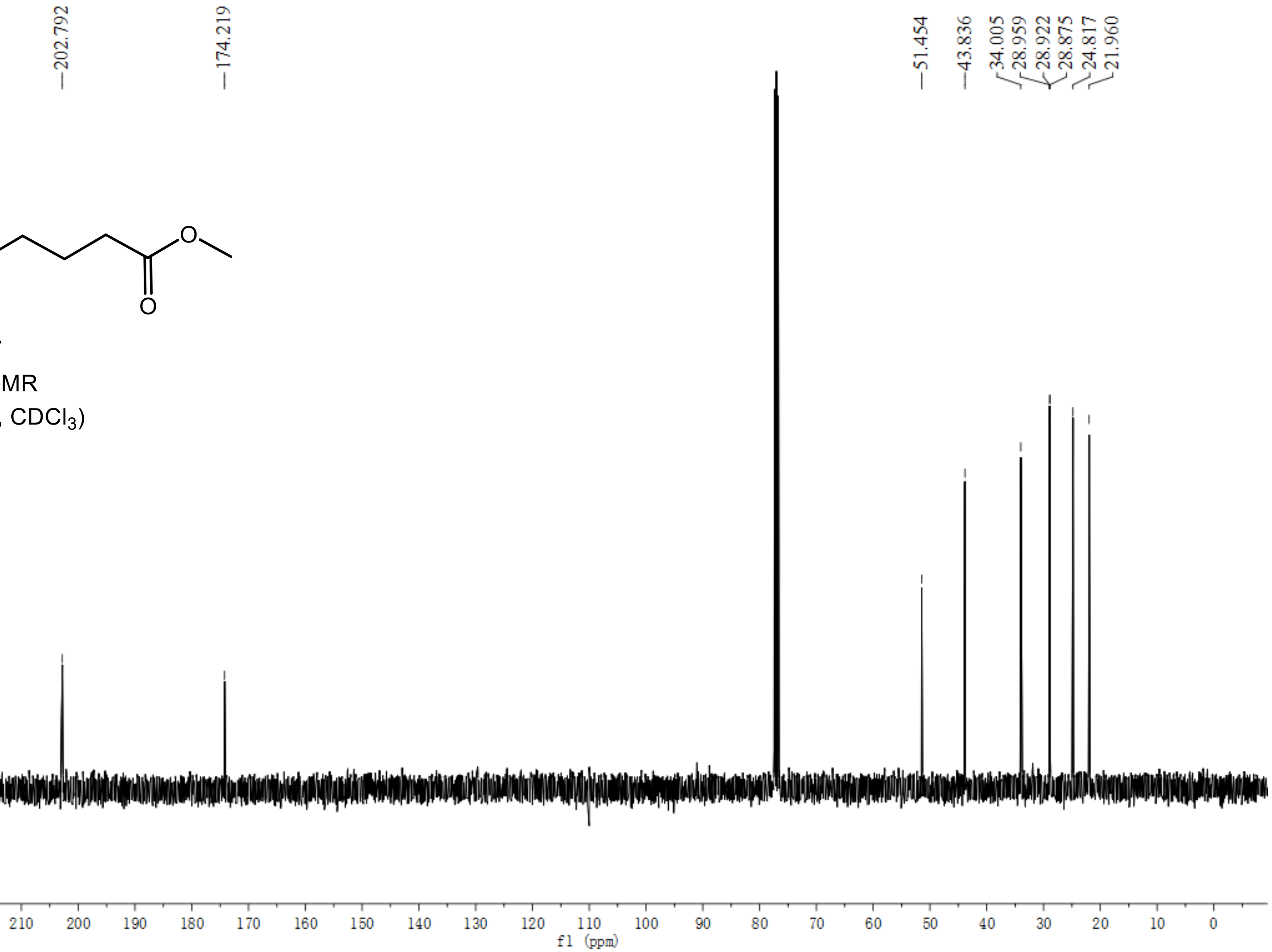

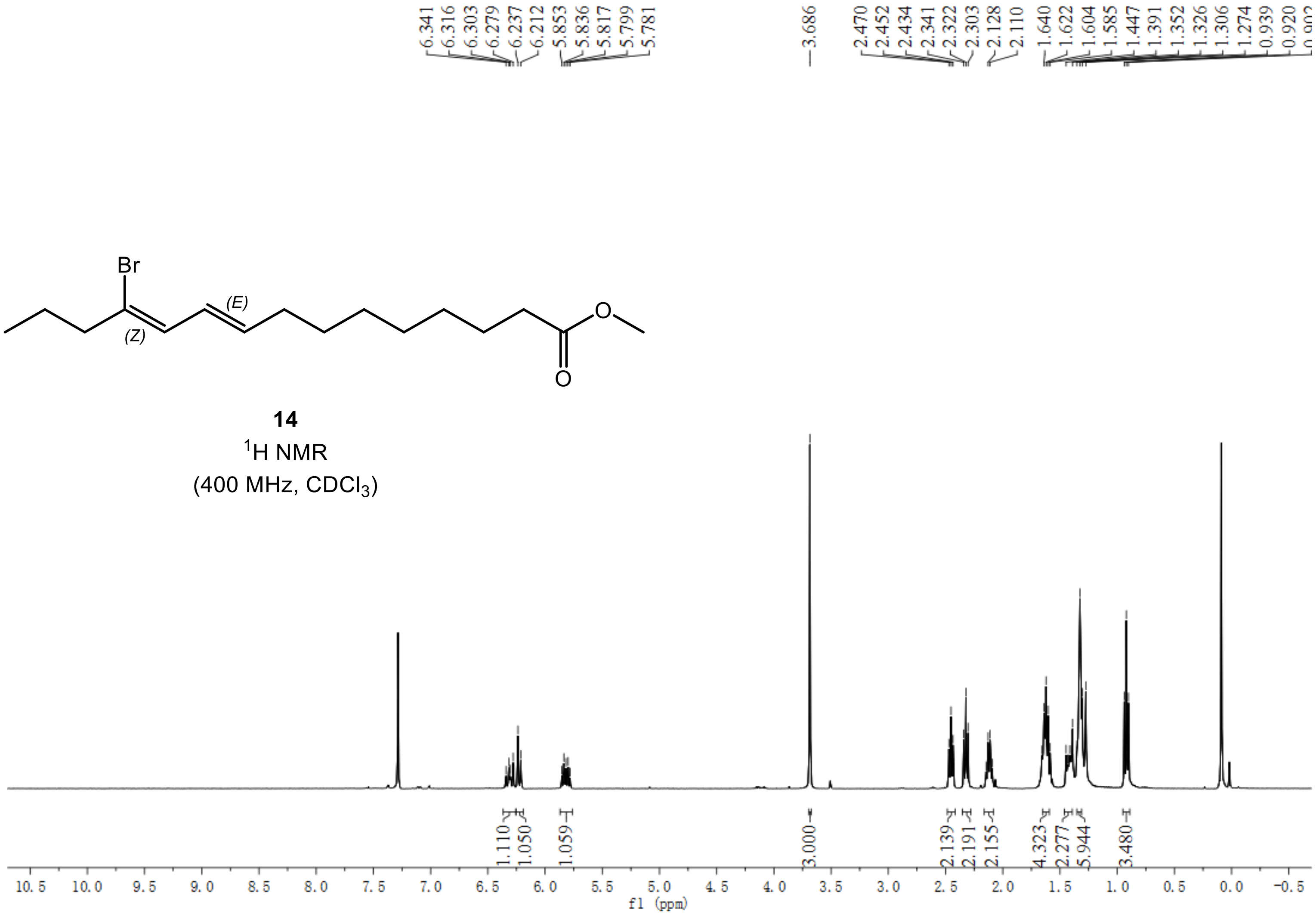

14

^{1}H NMR

(400 MHz, $CDCl_3$)

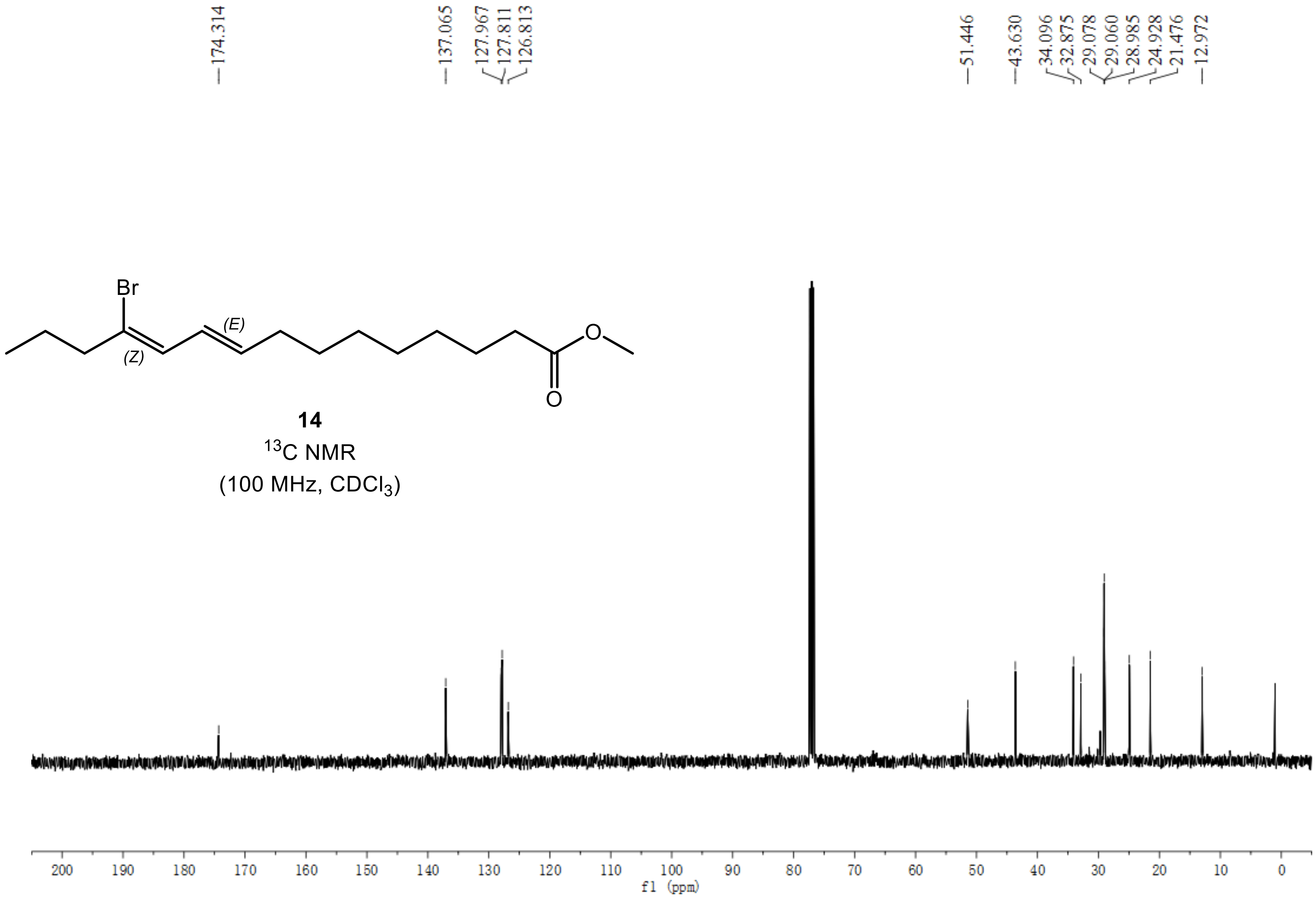

14

^{13}C NMR

(100 MHz, $CDCl_3$)

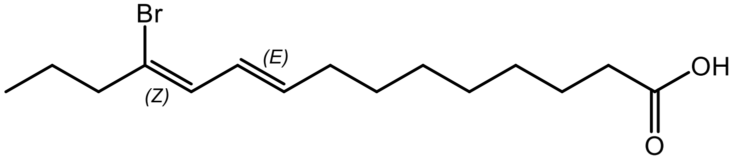

15
^{1}H NMR
(400 MHz, $CDCl_3$)

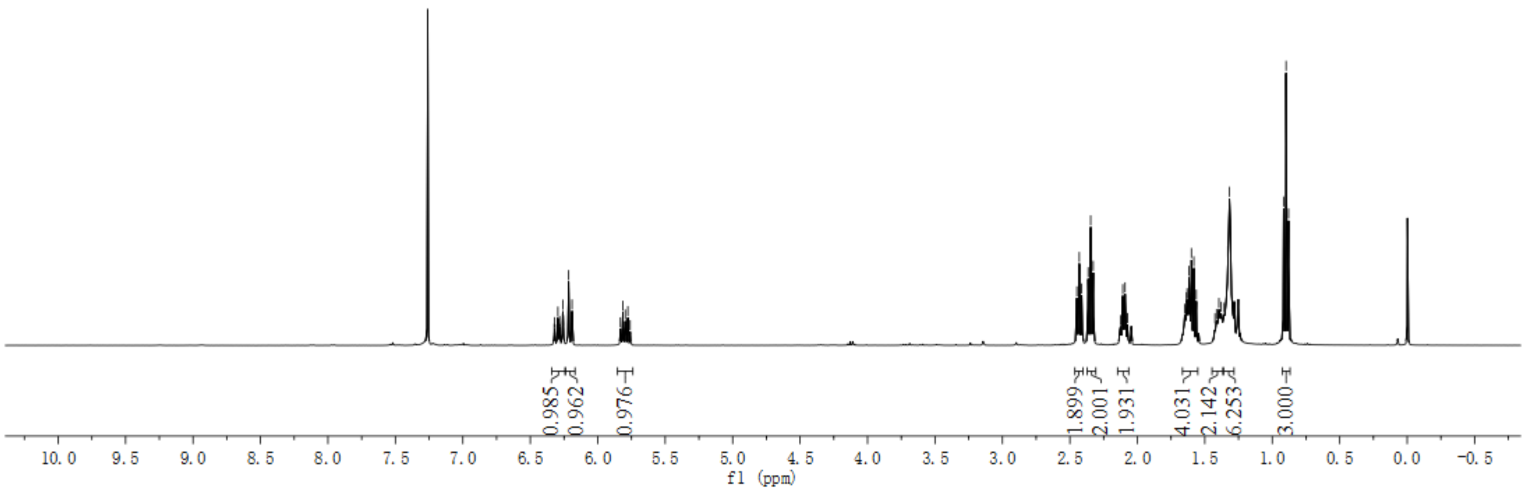

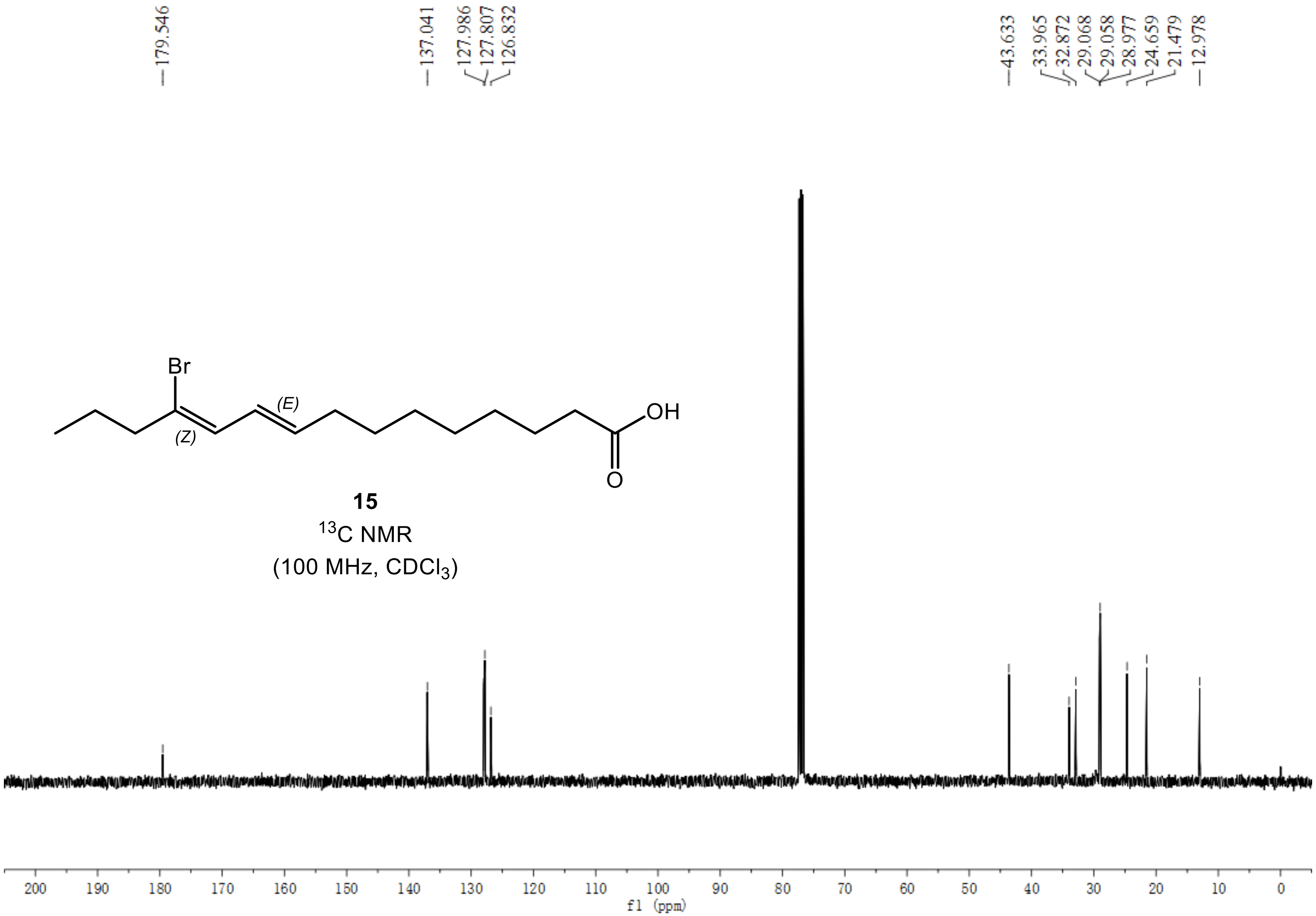
179.546
137.041
127.986
127.807
126.832
43.633
33.965
32.872
29.068
29.058
28.977
24.659
21.479
12.978
Br
(Z)
(E)
OH
O
15
^{13}C NMR
(100 MHz, $CDCl_3$)
f1 (ppm)

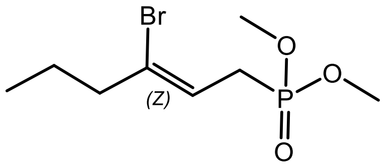

S5

^{1}H NMR

(400 MHz, $CDCl_3$)

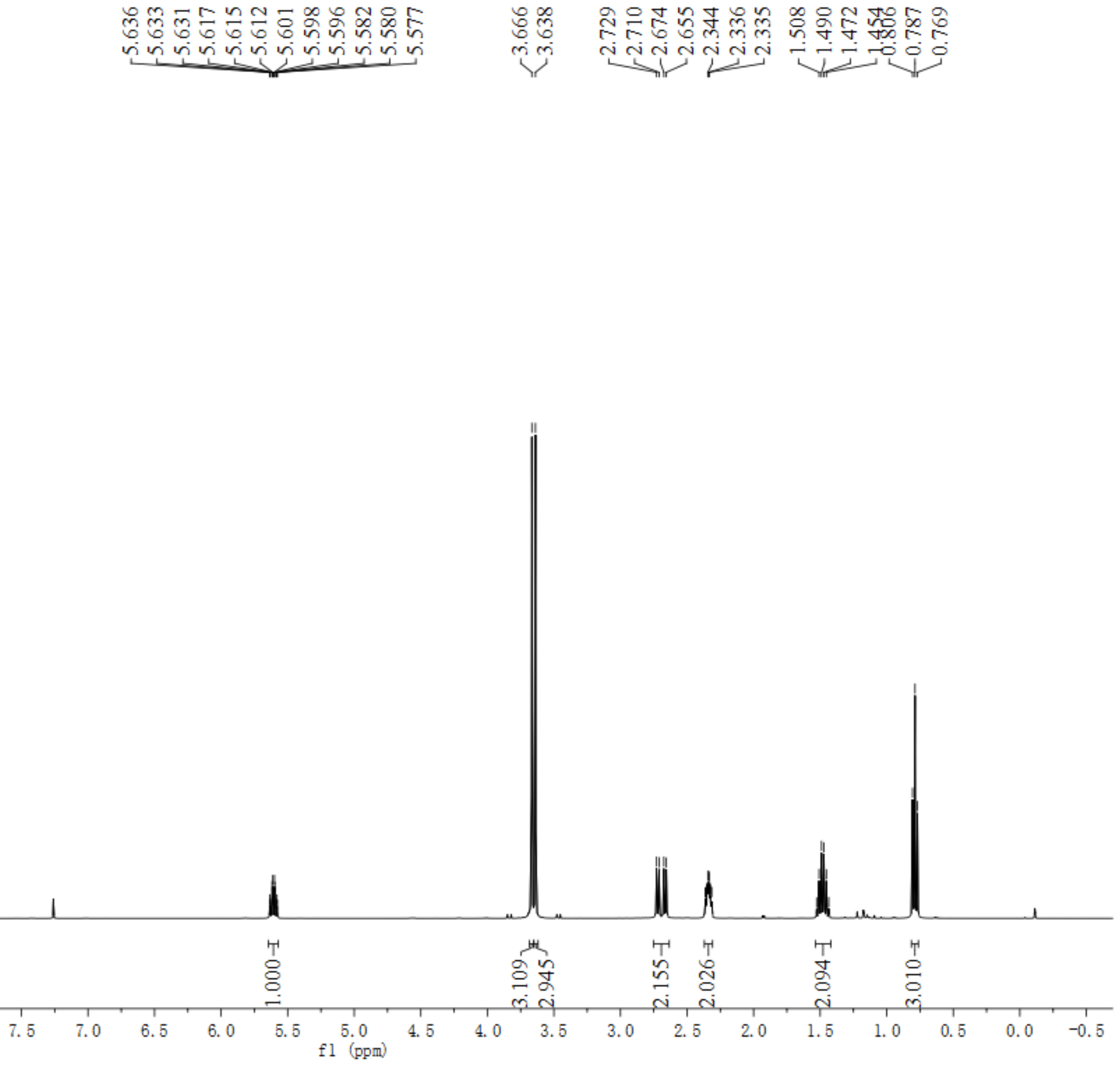

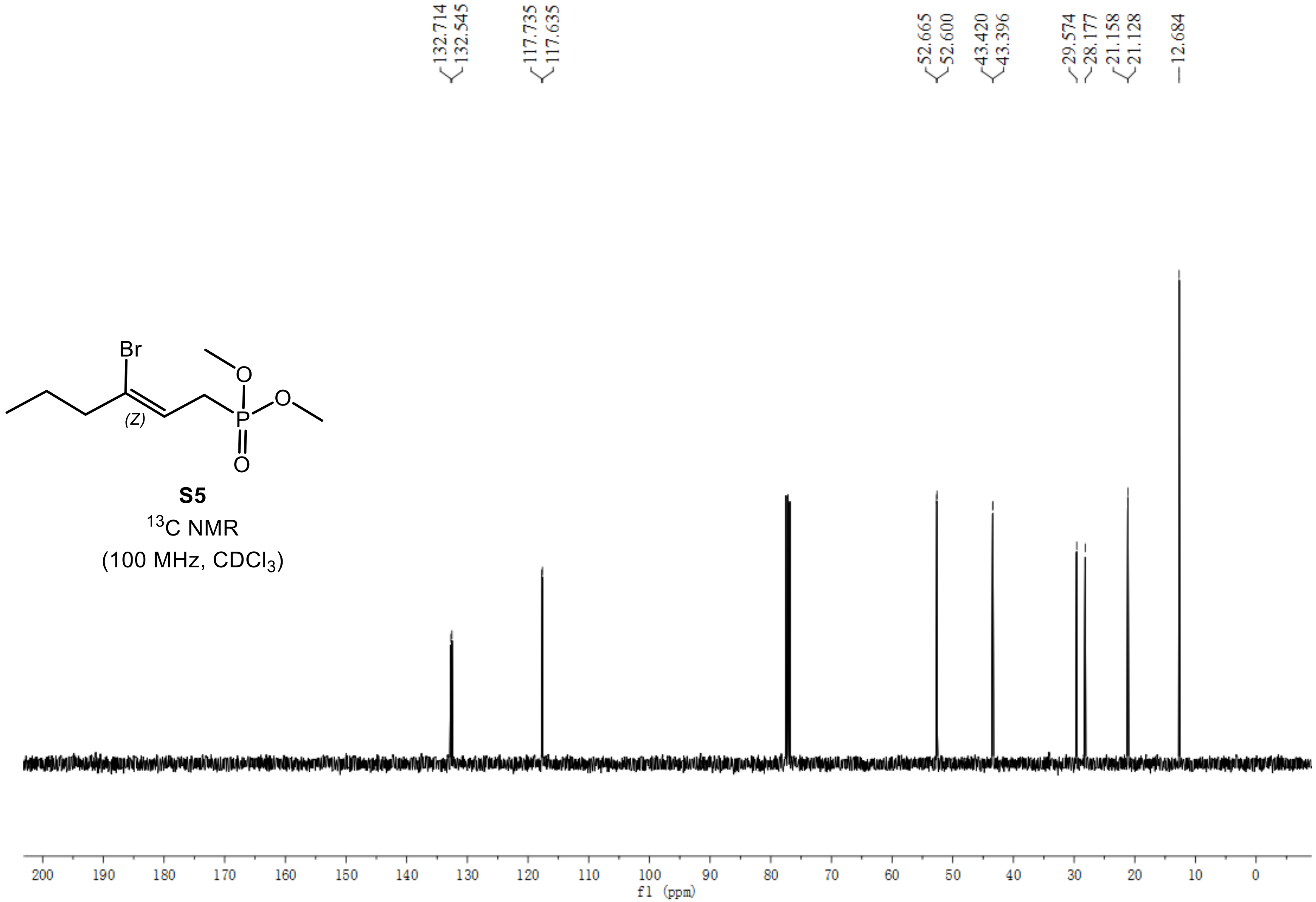

S5
^{13}C NMR
(100 MHz, $CDCl_3$)

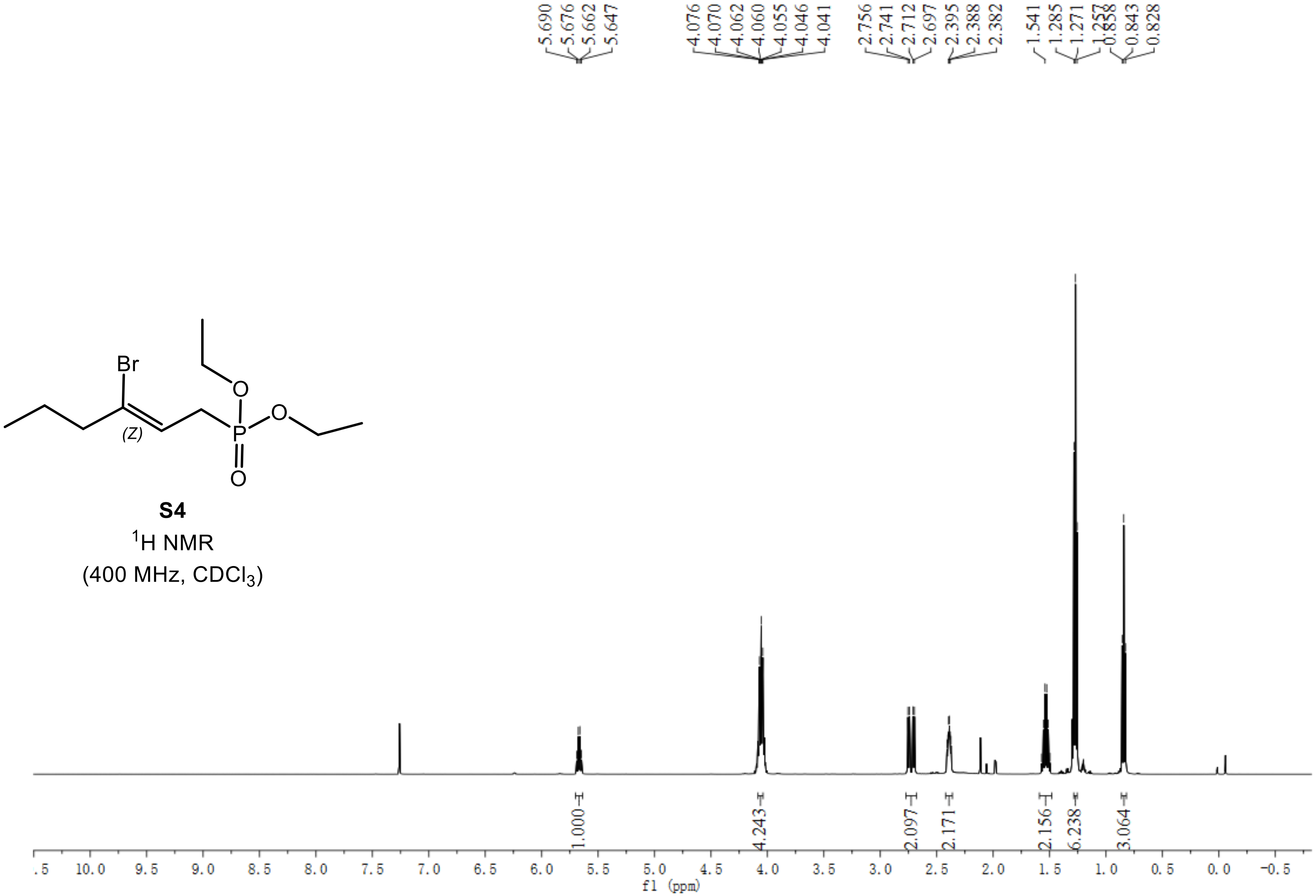

S4
^{1}H NMR
(400 MHz, $CDCl_3$)

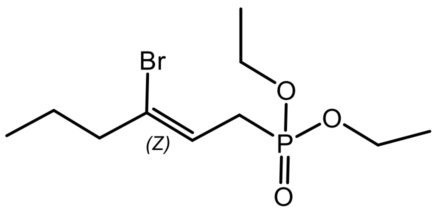

S4

^{13}C NMR

(100 MHz, $CDCl_3$)

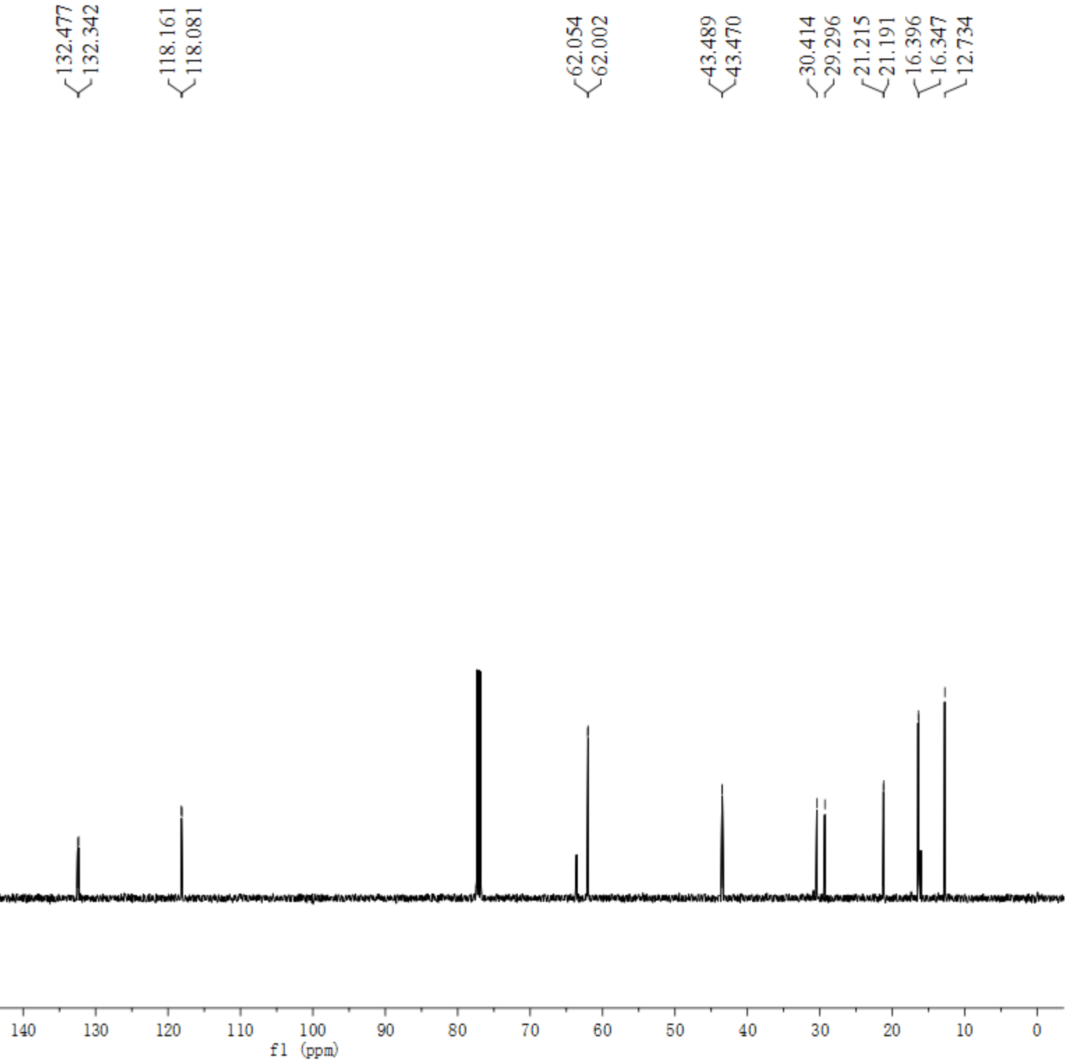

Supplement: Supplementary file 1 [file marinedrugs-19-00288-s001.zip › marinedrugs-1222549-supplementary.pdf]
